# A New Multidisciplinary Home Care Telemedicine System to Monitor Stable Chronic Human Immunodeficiency Virus-Infected Patients: A Randomized Study

**DOI:** 10.1371/journal.pone.0014515

**Published:** 2011-01-21

**Authors:** Agathe León, César Cáceres, Emma Fernández, Paloma Chausa, Maite Martin, Carles Codina, Araceli Rousaud, Jordi Blanch, Josep Mallolas, Esteban Martinez, Jose L. Blanco, Montserrat Laguno, Maria Larrousse, Ana Milinkovic, Laura Zamora, Neus Canal, Josep M. Miró, Josep M. Gatell, Enrique J. Gómez, Felipe García

**Affiliations:** 1 Infectious Diseases Unit, Hospital Clinic, Institut d'Investigacions Biomèdiques August Pi I Sunyer, University of Barcelona, Barcelona, Spain; 2 Bioengineering and Telemedicine Unit, Technical University of Madrid, Madrid, Spain; 3 Pharmacy Service, Hospital Clinic, Institut d'Investigacions Biomèdiques August Pi I Sunyer, University of Barcelona, Barcelona, Spain; 4 Clinical Institute of Psychiatry and Psychology, Hospital Clinic, Institut d'Investigacions Biomèdiques August Pi i Sunyer, University of Barcelona, Barcelona, Spain; 5 Health Economics and Outcomes Research, IMS Health, Inc., Barcelona, Spain; University of Toronto, Canada

## Abstract

**Background:**

Antiretroviral therapy has changed the natural history of human immunodeficiency virus (HIV) infection in developed countries, where it has become a chronic disease. This clinical scenario requires a new approach to simplify follow-up appointments and facilitate access to healthcare professionals.

**Methodology:**

We developed a new internet-based home care model covering the entire management of chronic HIV-infected patients. This was called Virtual Hospital. We report the results of a prospective randomised study performed over two years, comparing standard care received by HIV-infected patients with Virtual Hospital care. HIV-infected patients with access to a computer and broadband were randomised to be monitored either through Virtual Hospital (Arm I) or through standard care at the day hospital (Arm II). After one year of follow up, patients switched their care to the other arm. Virtual Hospital offered four main services: Virtual Consultations, Telepharmacy, Virtual Library and Virtual Community. A technical and clinical evaluation of Virtual Hospital was carried out.

**Findings:**

Of the 83 randomised patients, 42 were monitored during the first year through Virtual Hospital (Arm I) and 41 through standard care (Arm II). Baseline characteristics of patients were similar in the two arms. The level of technical satisfaction with the virtual system was high: 85% of patients considered that Virtual Hospital improved their access to clinical data and they felt comfortable with the videoconference system. Neither clinical parameters [level of CD4+ T lymphocytes, proportion of patients with an undetectable level of viral load (p = 0.21) and compliance levels >90% (p = 0.58)] nor the evaluation of quality of life or psychological questionnaires changed significantly between the two types of care.

**Conclusions:**

Virtual Hospital is a feasible and safe tool for the multidisciplinary home care of chronic HIV patients. Telemedicine should be considered as an appropriate support service for the management of chronic HIV infection.

**Trial Registration:**

Clinical-Trials.gov: NCT01117675.

## Introduction

Since the appearance of highly active antiretroviral treatments (HAART) the process of HIV/AIDS becoming a chronic disease in the industrialised world has led to a dramatic change in the illness paradigm [1]. Patients who would previously have been terminally ill are now chronically ill, and palliative care has become chronic care [2], [3]. This situation requires a completely new approach to care of the HIV/AIDS patient.

For patients, a chronic disease course means visiting their hospital every three months, first to perform a blood test, second for the follow-up appointment and, finally, to collect their medication from the hospital pharmacy. This clinical routine can interfere with patients' attempts to return to normality in their daily lives, and may create problems with employers due to work absences [4], [5]. In addition, patients need to be closely monitored in order to maximise their adherence to medication, and therefore prevent the development of resistance.

For the infectious diseases physician the follow-up of a chronic HIV patient has become easier, because these patients are relatively young, show few comorbidities and do not require complex monitoring, simply a blood test and routine appointments every three months to check on results.

However, there is still no cure for the infection and the number of chronic HIV-infected patients is increasing year by year, thereby placing greater demands on healthcare systems. As a result there is a need to optimise health resources, both in terms of infrastructure and staffing levels.

In this regard, ideas about how to approach this situation may be gained by looking at other chronic diseases such as diabetes [6]–[9], chronic obstructive pulmonary disease [10]–[12] or congestive heart failure [13]–[15], which have made use of telemedicine for several years now. Research has shown that a multidisciplinary management programme and home-based intervention can reduce hospital readmission rates and length of hospital stay in patients with chronic cardiac disease [16]–[18], as well as improving their quality of life [19]–[21]. In the case of diabetes, telemedicine allows the frequent transmission of blood glucose values to healthcare providers, thereby enabling them to modify the medical regime and/or diet so as to improve metabolic control [22]–[25].

Telecare involves the delivery of health and social care to individuals within the home or wider community, with the support of systems enabled by information technology [26]. It introduces new forms of assessment designed to improve the quality and variety of information which clinicians have about a patient's health status. Measures of functional status and quality of life, in addition to physiological monitoring, can be translated into accurate predictors of health risk, and they can be combined with electronic alarm systems to initiate an appropriate course of action. This information is invaluable in identifying and treating problems, sometimes at an earlier stage [27].

A further aspect is that the coordination of the care team and the involvement of patients in their own care [28] seem to be factors of great importance for good chronic disease management [29]. A multidisciplinary care team is also desirable in a disease such as HIV/AIDS, where psychological and social factors have an increasing influence on a patient's health status. Indeed, chronic care requires a holistic model that integrates doctors, psychologists, nurses, social workers and pharmacists into the same team, of which the patient should also be seen as a member [30].

Telemedicine is attractive to physicians because it offers them the possibility of reaching all those patients who need their advice regardless of distance, while the appeal to patients is that it gives them a greater access to health care. Naturally, both groups prefer the face-to-face consultation whenever possible, but consultations can sometimes be offered through a form of telemedicine, which is more convenient, cheaper, less time-consuming and may be equally satisfactory for both parties. Furthermore, telemedicine may reduce the patient's travelling time and absence from work, thereby decreasing the overall societal costs.

Given the current situation facing patients and health providers, and the enormous possibilities offered by the internet, we developed a telemedicine service to complement standard care. Specifically, this involved a telecare follow-up for stable HIV-infected patients in the chronic phase of their disease. The present study evaluate the home care provided by this telemedicine system called Virtual Hospital, comparing standard care received by HIV-infected patients with Virtual Hospital care. The objective of the study is to demonstrate that Virtual Hospital could improve and facilitate the access of the patients to the health system without any deleterious effect on their care. The technical structure of the system has already been published in several telemedicine journals [31], [32].

## Methods

An open-label, two-arm, prospective randomised study was performed between January 2005 and December 2007 in a university hospital in Barcelona. Participants were 3500 HIV outpatients. The protocol for this trial and supporting CONSORT checklist are available as supporting information; see Checklist S1 and Trial Protocol S1.

The main goals were the definition, development, clinical routine installation and evaluation of a telemedicine service that compares standard care with telecare follow-up for stable HIV-infected patients in the chronic phase of their disease. The study was approved by the local Human Research Ethics Committee. Patients signed written informed consent and were randomised (with balanced randomization [1∶1] through a computer generated randomization list without any restriction) to either: Arm I, where they were monitored virtually via the internet during the first year of follow-up; or arm II, where they were monitored in the day hospital via standard care. After one year of follow up, patients switched their care to the other arm.

Standard Care in a stable HIV infected adult consists firstly in to perform a blood test (to assess biochemical parameters, CD4 T cells, HIV-1 viral load) 15 days before the day of consultation in the Infectious Diseases Day Care Hospital (laboratory needs 2 weeks to have all the results of the blood test performed). Secondly, the infectious disease physician during consultation, checks the analysis, asks about any clinical change and expends antiretroviral prescriptions if the patient is treated. Thirdly, patient goes with the antiretroviral prescription to the hospital Pharmacy to perform a visit with the pharmacist to check antiretroviral compliance and finally to collect the drugs. The patient repeats this clinical routine every 3–4 months.

The telecare system, Virtual Hospital, offered integrated online patient monitoring by a multidisciplinary care team (nine infectious diseases specialists, three nurses, one psychologist, one psychiatrist, three pharmacists and one social worker), allowing the patient to be remotely followed-up by these professionals and have access to their own clinical and pharmaceutical files. See test web http://web.gbt.tfo.upm.es/clinic/login.asp.

Inclusion criteria were stable patients (CD4 >250 cells/mm^3^ during at least the three months prior to inclusion to the study) who had access to a computer and to broadband. No other specific demographics (risk groups, educational level and nationality) or clinical characteristics (hepatitis B or C coinfection) were considered selection criteria. Exclusion criteria were HIV-infected adults with current therapeutic failure (defined by detectable viral load on treatment or CD4 cell count <250 cells/ml), tumours, opportunistic infections, or pregnant women.

**Figure 1:**
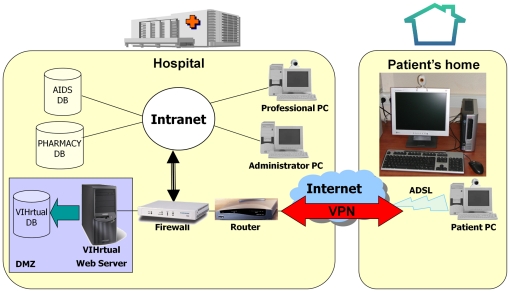
Architecture of the Virtual Hospital. The architecture of this web-based system has two parts. The first was the hospital infrastructure, where we added our server to the existing demilitarised zone (DMZ) of the hospital, which was protected by a firewall and integrated into the hospital's information system network. Health professionals accessed this server via the hospital's intranet. The second part was the home infrastructure, where the patient accessed the server via a basic broadband connection and, for security reasons, through a virtual private network (VPN).

The architecture of this web-based system had two parts. The first was the hospital infrastructure, where we added our server to the existing demilitarised zone (DMZ) of the hospital, which was protected by a firewall and integrated into the hospital's information system network. Health professionals accessed this server via the hospital's intranet. The second part was the home infrastructure, where the patient accessed the server via a basic broadband connection and, for security reasons, through a virtual private network (VPN) (Figure 1). Of critical importance in the system was the connection of the server with three databases. The Virtual database was the new database created for the telemedicine system, where the data of patients involved in the trial were stored. This database was filled and synchronised with the HIV/AIDS database, which the Infectious Diseases Service of the Hospital Clinic has been using over the last twenty years; this database includes the records of over 5,000 HIV/AIDS patients. Finally, the server was also connected to the pharmacy database, where all the available drugs and data related to antiretroviral compliance were recorded.

The graphical interface was carefully designed in order to make it user friendly for both professionals and patients. Another main goal was to develop a low-cost system so as to enable an increased number of patients to be offered such care in the future. This is why low-cost, home web cams and broadband were some of the chosen technologies for the implementation. Security was one of the most carefully designed aspects of the project, mainly because of its experimental nature and due to the characteristics of the disease in question. As well as securing the communications, via VPN tunnelling, patient data were also encrypted and all personal identification data were removed. A completely separate tool was developed outside the web system, so that only the system administrator would have access to it. All access to the system was monitored, and the system automatically sent an alert e-mail to the technical manager in the event of recurrent access. More technical information about Virtual Hospital has been previously reported [31], [32].

Virtual Hospital offered four main services*:* Virtual Consultations, Telepharmacy, Virtual Library and Virtual Community.

**Figure 2:**
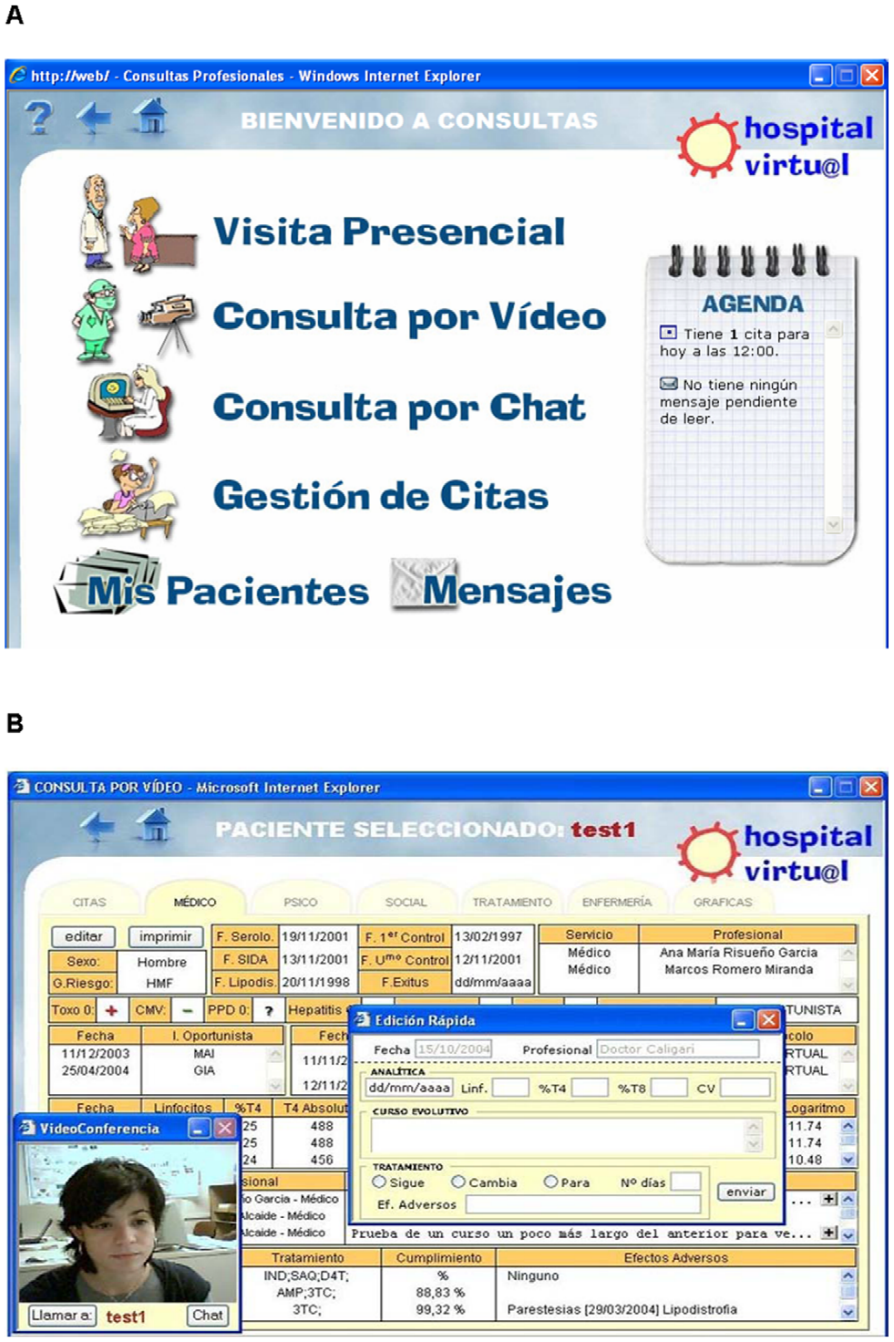
Electronic Health Record. A. This figure shows the Virtual Consultation menu, where users select appointments conducted via standard care, videoconferencing, chat sessions or message exchanges. An electronic diary is also available so that at the end of the appointment, the patient and the professional could set a time for the next one. B. Here it shows an example of a teleconference appointment. Electronic Health Record contains demographic, epidemiological and clinical data (Hepatitis B or C coinfections; CD4 cell count and HIV-1 plasma viral load evolution; HAART-compliance and tolerance; comments of the infectious diseases physician) and is available to both professionals and patients during any of the sessions.

*Virtual consultations* had two levels: first, appointments/consultations conducted via videoconferencing; and second, chat sessions or message exchanges for emergency or off-schedule consultations. During any of these sessions the Electronic Health Record was available to both professionals and patients (see Figure 2). However, whenever patients wish it, in addition to the virtual consultations they might have an in-person consultation with the health professionals. It should be noted that psychological and social data were also integrated into the patients' records. An electronic diary was also available so that at the end of the appointment, the patient and the professional could set a time for the next one.

**Figure 3:**
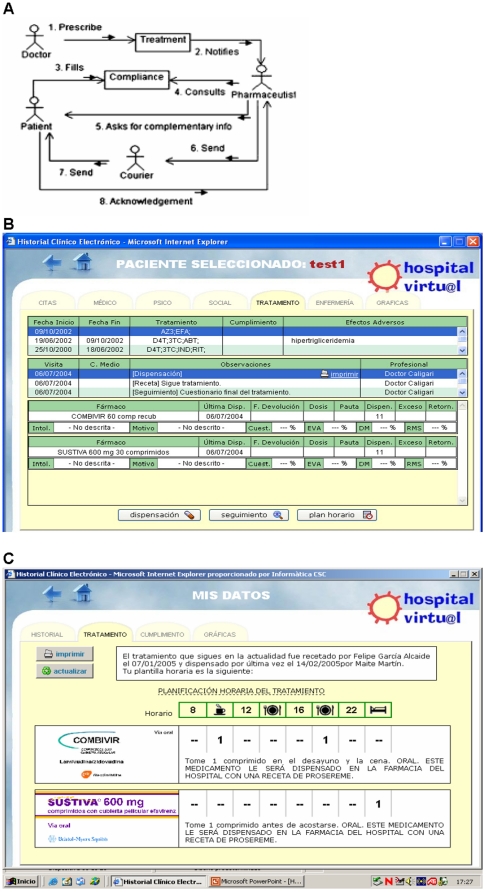
Telepharmacy. A. Telepharmacy allows the pharmacist to receive electronic prescriptions, to perform virtual consultations about compliance, adverse events or interactions, and to send the antiretroviral medication to the patient's home by courier. B. This figure shows how professionals track patient's HAART-history. C. In this section, the patients can also visualise the evolution of their own treatments on charts and consult basic information on the antiretroviral drugs available.

*Telepharmacy* allowed the pharmacist to receive electronic prescriptions, to perform virtual consultations about compliance, adverse events or interactions, and to send the antiretroviral medication to the patient's home by courier. The standard care in our centre is that patients take their antiretroviral prescriptions to the hospital pharmacy, where the pharmacist sees the patient, checks whether he/she is having any problems with treatment, and dispatches the drugs (in Spain antiretroviral drugs are given out exclusively in hospital pharmacies). The telepharmacy system enabled patients to track the evolution of their treatment on charts and consult basic information regarding the available antiretroviral drugs. The new process and the telepharmacy system are shown in Figure 3.

*Virtual library* stored validated information about HIV as links to other web pages, for both patients and professionals. All links were categorised by their type of source and were included in different groups according to the subject they referred to.

*Virtual community* provided space to exchange information about the disease and the project, as well as to share opinions or comment on articles and news items. The information offered by this tool differed according to the user (i.e. healthcare professional or patient). Exclusive to professionals was the clinical session option, where they could share opinions about the cases they were responsible for at the time.

A brief twenty-minute introduction to the system's functioning was offered to both professionals and patients prior to starting the study. An external company was hired to help patients with any technical problems involving hardware or software (patients had a telephone number to call in such cases). This company resolved most of the problems by telephone, but when necessary a technician went to the patient's house.

The technical performance of Virtual Hospital was evaluated by both professionals and patients. Validated questionnaires were used to assess different aspects of the system, with items being rated on a five-point scale, from 1 for the most negative appraisal (“Not appropriate/Totally disagree”) to 5 for the most positive one (“Very appropriate/Totally agree”). Parameters regarding access, organisation of the system, the need for training, reliability, usability, acceptance, usefulness and satisfaction were also evaluated.

The Virtual Hospital was also evaluated in terms of its clinical performance, assessing the impact on HIV clinical parameters [CD4 cell count, viral load (VL), opportunistic infections, death], the need to start combined AntiRetroviral Treatment (cART) and cART-compliance throughout the study follow-up. Adherence was evaluated at each clinical consultation by monitoring pharmacy refills and through self-reports and was considered high if the patients take more than 90% of the scheduled medication [33].

Quality of life was evaluated through a questionnaire that has been validated in HIV patients, the Mini International Neuropsychiatric Interview (MINI) [34], [35]. This assessed various aspects including mental and physical health, cognitive and social functioning, energy levels and vitality, perceived social support, sleep and sexuality. Scores were standardised on a scale from 0 to 100, where 0 corresponds to better quality of life and 100 to a worse one.

Psychological and emotional Impact was evaluated using several validated screening questionnaires: the Hospital Anxiety and Depression (HAD) Scale [36], [37] to measure anxiety and depression, Goldberg's General Health Questionnaire (GHQ-28) [38], [39] to screen for symptoms of psychopathology, and the Psychosocial Adjustment to Illness Scale (PAIS) [40] to measure the impact of a medical condition on general psychosocial functioning.

A qualitative statement about health services utilization in the study population, was registered in one of the questionnaires that patients field. There were two questions (question 7 and 9) addressing this issue. Question 7 was: How many times per year, do you consult the following health care providers? (7.1–7.3 Infectious Diseases Unit, 7.4 Pharmacy, 7.5 Social Work, 7.6 Psychiatry, 7.7 Primary Care, 7.8 Others). The answers were: never, one or two per year, each three or four months, each two months, each month, more than once per month. Question 9 was: How often has consulted to the following health care providers in the last three months? (Excluding Virtual Consultations) (9.1–9.3 Infectious Diseases Unit, 9.4 Pharmacy, 9.5 Social Work, 9.6 Psychiatry, 9.7 Primary Care, 9.8 Others). The answers were: never, ever, several times, once a month, more than once a month.

Quantitative characteristics were described as medians and the interquartile range (IQR), while qualitative data were given as frequencies and percentages. Laboratory parameters were compared using a Wilcoxon signed-rank test. Intra-individual changes in these parameters were compared for the period monitored by Virtual Hospital versus the period involving standard care (intra-arm or intra-individual variations), and variability between arms at the end of the two-year follow-up was also compared (inter-individual variations). All statistical analyses were performed using SPSS version 15.0.1. Patients were analyzed in the groups to which they were randomly assigned and a per-protocol analysis was conducted.

Sample size calculations (using McNemar's test of paired proportions with a significance level of 5%) showed that 77 patients would have 80% power in detecting differences of 25% when the expected proportion of discordant pairs was 35%. The selection of this difference is consistent with other studies that found that non-adherence was associated with poorer virological outcomes [41]–[44]. With an estimated lost-to-follow-up rate of 10%, the total sample size required would be 90 patients.

## Results

**Figure 4:**
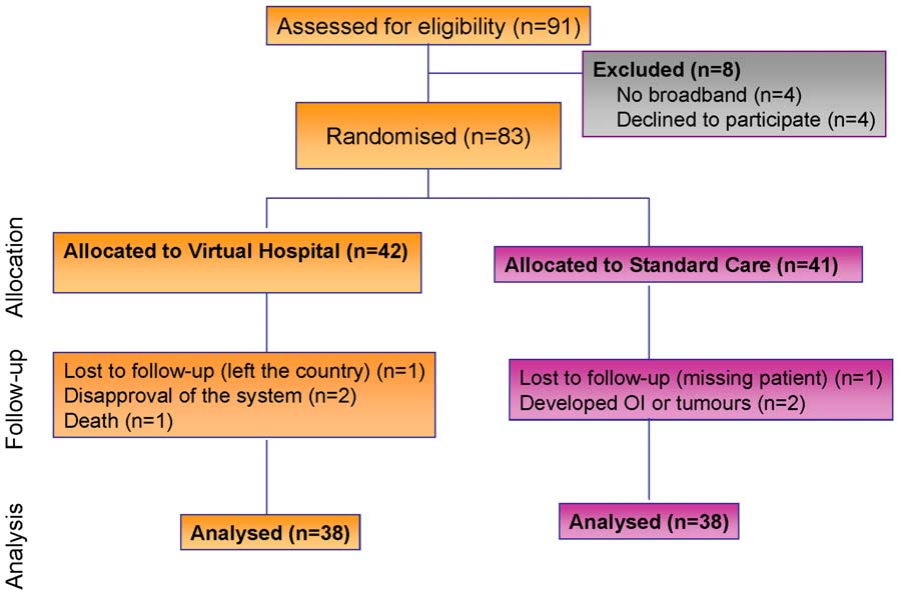
Patient availability. 91 patients were assessed for eligibility, but four were excluded due to a lack of broadband, while a further four declined to participate. Of the remaining 83 randomised patients, 42 were monitored during the first year through Virtual Hospital (Arm I) and 41 through standard care (Arm II). Patients switched their care arm in year two of the study. Over the two years of follow-up, seven patients (8%) discontinued the study: two were lost to follow-up (one left the country and one was whereabouts unknown), two left the Virtual Hospital because they disapproved of the system, two developed tumours (one non-Hodgkin lymphoma and one hepatocellular carcinoma), and one patient was killed in a traffic accident. Overall, 76 patients (92%) completed the study.

Initially, 91 patients were assessed for eligibility, but four were excluded due to a lack of broadband, while a further four declined to participate. Of the remaining 83 randomised patients, 42 were monitored during the first year through Virtual Hospital (Arm I) and 41 through standard care (Arm II). Patients switched their care arm in year two of the study. Baseline characteristics of the patients were well-balanced between the arms (see Table 1). Overall, 93% (n = 77) of the cohort had an active working life and 38% (n = 32) had a university degree. A similar number of patients (n = 46, 55% of the overall cohort) were receiving antiretroviral therapy at baseline (see Table 1). Over the two years of follow-up, seven patients (8%) discontinued the study: two were lost to follow-up (one left the country and one was whereabouts unknown), two left the Virtual Hospital because they disapproved of the system, two developed tumours (one non-Hodgkin lymphoma and one hepatocellular carcinoma), and one patient was killed in a traffic accident (see patient availability in Figure 4). Overall, therefore, 76 patients (92%) completed the study.

**Table 1:** Baseline characteristics of the patients.

CATEGORY	SUBCATEGORY	Cohort	Arm I	Arm II	P
**N**		83	42	41	
Age* (years)		37 (34–43)	37 (34–43)	37 (34–43)	0.9
**Gender** **	**Male**	72 (87)	36 (86)	36 (88)	0.56
	**Female**	11 (13)	6 (14)	5 (12)	
**Risk Group** **	**HMS**	56 (68)	25 (60)	31 (76)	0.36
	**HTS**	20 (24)	13 (31)	7 (17)	
	**DU**	7 (8)	4 (9)	3 (7)	
**Nationality** **	**Spanish**	66 (79)	31 (74)	35 (86)	0.20
	**Other**	17 (21)	11 (26)	6 (14)	0.20
**Educational level** **	**Primary school**	10 (12)	5 (12)	5 (12)	0.97
	**Secondary school**	41 (49)	20 (48)	21 (51)	0.12
	**University**	32 (38)	17 (40)	15 (37)	0.15
**Employment status** **	**Unemployed**	6 (7)	3 (7)	3 (7)	0.97
	**Working**	77 (93)	39 (93)	38 (93)	0.12
	**Disabled**	0	0	0	0.15
**Hepatitis C** **		10 (12)	5 (12)	5 (12)	0.72
**Hepatitis B** **		9 (11)	3 (7)	6(15)	0.17
CD4+ T cells (cells/mm3)*		602 (471–726)	585 (461–738)	636 (511–718)	0.66
**Log_10_VL (copies/ml)** ****		2.79 (0.14)	2.79 (0.22)	2.90 (0.20)	0.40
**Treatment** **		46 (55)	24 (57)	22 (54)	0.52

P for comparison between arm I and II. *Median (IQR),

**(n, %),

***(n, % of patients on treatment),

****Mean (SE). HMS: Homosexual. HTS: Heterosexual. DU: Drug users.

### Technical evaluation

Questionnaires submitted by patients (n = 76) revealed a high level of technical satisfaction with the system after one year of Virtual Hospital care (Table 2). Overall, 85% of patients (n = 65) considered that Virtual Hospital improved their access to clinical data, as compared with standard care, and they felt comfortable with the videoconference system. The system was rated by 82% (n = 62) of patients as an easy way to communicate among users. The same questionnaires were also completed by professionals (n = 18), with similar results (data not shown).

**Table 2:** Technical evaluation by patients of the Virtual Hospital.

CATEGORY	SUBCATEGORY	Patients*
**N**		76
**Access**	*The system meets my needs*	84
	*The data are easy to manage*	88
	*It is easy to input data*	78
	*The data are presented in a good way*	82
	*It is easy to correct the data*	65
	*Managing the data is quick*	65
	*The system improves access to clinical data*	85
**Organisation**	*The appointments process works well*	82
	*The system for dispatching medication works well*	64
	*The virtual library is useful*	67
	*The virtual community is useful and easy to use*	50
	*I feel comfortable with the videoconference system*	85
	*The system improves communication between users*	82
**Training**	*The period and type of training has been useful*	80
	*I have needed to consult additional documentation about the system*	17
	*I learned easily to use the system*	81
**Reliability**	*Virtual Hospital is secure and stable*	83
	*I have needed technical support*	15
	*Technical problems were resolved quickly*	73
	*I have lost data due to system errors*	5
**Usability**	*Navigating around the system is quick*	93
	*Collaboration between health workers has changed*	50
**Acceptance**	*The system is acceptable*	81
**Usefulness**	*The system is useful*	84.6
**Satisfaction**	*I am satisfied with Virtual Hospital*	69
	*I feel comfortable with the system*	90
	*I like Virtual Hospital*	86
	*Virtual Hospital improves my daily life*	74
	*I would like to use the system in the future*	85
	*The relationship with healthcare professionals is easier*	68
	*Virtual Hospital enables me to save time and money*	85

Questionnaire items were rated from 1 (most negative) to 5 (most positive).

*Proportion of patients who gave the two highest ratings (4 =  “appropriate/agree”, or 5 =  “Very appropriate/Totally agree”).

### Clinical evaluation

No opportunistic infections were reported. Regarding the immunologic evaluation there were no intra-individual (data not shown) or inter-individual differences over time in CD4 cell counts as monitored through Virtual Hospital versus standard care (See Table 3).

**Table 3:** Study findings.

CATEGORY	SUBCATEGORY	Cohort	Arm I	Arm II	P
**N**		83	42	41	

CLINICAL EVALUATION	SUBCATEGORY	Cohort	Arm I	Arm II	P
**CD4+ T cells (cells/mm3)** *	**Month 12**	712 (32)	738 (46)	796 (50)	0.4
	**Month 24**	751 (38)	688 (43)	706 (58)	0.2
**Log_10_VL (copies/ml)** *	**Month 12**	2.76 (0.13)	2.58 (0.19)	2.40 (0.19)	0.3
	**Month 24**	2.57 (0.14)	2.92 (0.20)	2.74 (0.21)	0.4
**Need to start HAART** **	**Month 12**	3 (4)	1 (1)	2 (2)	
	**Month 24**	3 (4)	0 (0)	3 (4)	
**cART switching** **	**Month 12**	16 (19)	8 (10)	8 (10)	
	**Month 24**	7 (8)	4 (5)	3 (4)	
**cART compliance** *	**Month 12**	93.4 (1.2)	94.5 (1.5)	95.1 (1.2)	0.3
	**Month 24**	94.5 (0.9)	91.9 (1.9)	93.6 (.3)	0.4
**Tumors** **	**Month 12**	2 (2)	0 (0)	2 (2)	
	**Month 24**	0 (0)	0 (0)	0 (0)	
**Deaths** **	**Month 12**	1 (1)	0 (0)	0 (0)	
	**Month 24**	0 (0)	0 (0)	0 (0)	
**Quality of life assessment** **	**Month 12**	29.0 (2.3)	30.4 (3.4)	32.0 (3.8)	0.5
	**Month 24**	32.1 (2.6)	27.2 (2.6)	32.1 (3.7)	0.9

PSYCHOLOGICAL EVALUATION	SUBCATEGORY	Cohort	Arm I	Arm II	P
**HAD scale** ***	**Month 12**	17.2 (0.4)	18.1 (0.6)	16.0 (0.5)	0.1
	**Month 24**	17.5(0.6)	16.9 (1.0)	18.3 (0.8)	0.2
**Goldberg Questionnaire** ***	**Month 12**	4.9 (0.9)	5.9 (1.6)	3.6 (1.0)	0.8
	**Month 24**	5.6 (1.3)	5.9 (1.8)	5.4 (1.8)	0.6
**PAIS scale** ***	**Month 12**	66.5 (0.7)	67.3 (0.9)	66.7 (0.9)	0.5
	**Month 24**	66.4 (1.1)	67.0 (0.9)	65.8 (2.0)	0.8

HEALTH SERVICES UTILIZATION	SUBCATEGORY	Cohort	Arm I	Arm II	P
**Infectious Diseases Unit** ***	**Month 12 (4M/6M)**	78%/19%	78%/17%	70%/20%	0.6
	**Month 24(4M/6M)**	79%/18%	79%/18%	67%/29%	
**Pharmacy** ***	**Month 12 (4M/0)**	60%/33%	49%/32%	59%/34%	0.1
	**Month 24 (4M/0)**	59%/34%	48%/33%	58%/37%	
**Primary Care** ***	**Month 12 (0/1–4)**	45%/43%	45%/42%	43%/46%	0.3
	**Month 24 (0/1–4)**	45%/44%	45%/42%	42%/46%	
**Other health providers** ***	**Month 12 (0/1–2)**	65%/24%	51%/32%	73%/20%	0.3
	**Month 24 (0/1–2)**	64%/24%	51%/33%	75%/17%	

P for comparison between arm I and II.

*Mean (SE).

**(n, %). cART compliance: 93.4 means that patients took the 93.4% of the scheduled medication.

***(%). 4M: every 4 months. 6M: twice a year. 0: Never used. 1–4: Used from 1 to 4 times a year. 1–2: Used from 1 to 2 times a year.

Changes in viral load were also similar within individuals (data not shown) and between arms when comparing the periods for Virtual Hospital and standard care (See Table 3). Patients on treatment continued to have an undetectable viral load throughout the study period in both arms except 1 patient in arm II.

Twenty-three patients (28%) switched antiretroviral therapy during the study follow-up. Therapeutic changes were due to: simplification (in 15 patients); because of side events related to medication (in 6 patients); virologic failure (in 1 patient) and in 1 patient because of interrupted manufacture of the antiretroviral being taken.

6 patients (7%) started cART throughout the study. cART switching and the need to start cART was similar between arms (Table 3).

cART-compliance was high (more than 90%) and was similar within individuals (data not shown) and between arms during the two years of follow-up (Table 3).

### Quality of life and Psychological and emotional evaluation

Quality of life assessment did not shown any difference between arms during the two years of the study (Table 3). In addition, none of the psychological tests performed (HAD scale, Goldberg Health Questionnaire, Psychosocial Adjustment to Illness Scale) observed differences between arms during follow-up (Table 3)

### Health services assessment

Regarding the consultation to the health care providers (n = 76), most of the patients (78%, n = 59) performed a consultation every four months to the Infectious Disease Unit and most of the remaining patients twice a year. About sixty percent of patients (n = 46) consulted Pharmacy every 4 months and 33% (n = 25) of the patients never did it (those who are untreated). 76% (n = 58) of the patients never used Psychiatry services. The utilization of these stable patients to the Social Work services was very low. Primary care physicians and other health care providers were never consulted by 45% (n = 34) and 64% (n = 49) of the patients, respectively (See Table 3). There were no differences in health services utilization between study arms during study follow-up. Excluding Primary care, all the consultations performed not only infectious diseases specialists, but pharmacists, social worker and psychiatrists are integrated in the multidisciplinary care team of Virtual Hospital, allowing the patient to be remotely followed-up by these professionals too.

On the other hand, analyzing the health services utilization *out of* Virtual Consultations 69% (n = 52), 74% (n = 56) and 86% (n = 65) of the patients always consulted the Infectious diseases Unit, Pharmacy and Psychiatry services, respectively, using the virtual system. Again, similar results were observed between study groups.

### Costs Evaluation

Regarding budget to develop this telemedicine system, there were costs related to equipment in the hospital infrastructure. Hardware and software Virtual Hospital development cost was 50.000 euros. An external company was hired to help patients with any technical problems involving hardware or software installation, training or maintenance of the system with a cost of 70 euros per patient/year. Posting service cost, used eventually for medication delivery to patient home, was about 50 euros per patient/year. Home infrastructure (computer and broadband connection) was supported by patients.

## Discussion

We report the results of a prospective randomised study using a new model of multidisciplinary home care for chronic and stable HIV patients. The approach is based on a telemedicine platform, the Virtual Hospital, which covers the entire process of patient care (consultations, medical care, psychological and social support, medication, quality of life) via the internet and allows patients access to their own file. The study shows that Virtual Hospital constitutes a feasible, fairly satisfactory and safe tool for the clinical care of stable HIV-infected patients, and it has no deleterious effect on HIV clinical parameters, antiretroviral compliance, quality of life, or psychological and emotional status.

Patients were very satisfied with the system, as it has been showed by the satisfaction questionnaire and the low rate of withdrawal [76 from the 91 patients randomized ended the study (83%)]. In addition, when this study ended (december 2007), patients involved in it express their willingness to continue on Virtual Hospital and from then until now Virtual Hospital is being used as a routine work tool of the Infectious Disease Unit of the Hospital. Nowadays, there are round 200 patients cared of their HIV infection with such internet service. Otherwise being cared via Virtual hospital has not increased health resources utilization because of most of patients (69%, n = 52), out of Virtual consultations has never consulted to the Infectious diseases Unit even though almost a thirty percent of the study population (23 patients, 28%) has switched their antiretroviral medication, certainly one of the more difficult points in HIV management.

Telemedicine in HIV has been assessed in low-resource settings, both as an additional tool to help in the routine care of HIV-infected patients and for the training and education of healthcare providers [45]. Teleconferences, calls by mobile phones and e-health systems have all been used to support comprehensive HIV treatment programmes. In developed countries, telemedicine has been used as a tool to solve communication problems, as a prevention strategy for gay men [46] or special populations such as Latinos [47] or African-Americans [48], and to improve health outcomes including antiretroviral adherence [49], access to care for HIV-infected prisoners [50], clinical monitoring of highly treatment-experienced HIV-infected patients [51], cardiovascular risk assessment electrocardiograms at distance [52], and smoking cessation interventions [53]. The main innovation of our web-based system is that, to our knowledge, this is the first time that a telemedicine approach has enabled the whole range of HIV care to be offered to stable and chronic patients.

New procedures and improvements in telemedical patient education could support self-management and self-care of this disease through tools such as access to electronic medical records, availability of constant information updates, and the development of a custom online community of patients. The system is also scalable for future functionality and enables the integration of both information and services, which could facilitate cooperation between different professionals (primary and specialist care), creating units to improve the care of HIV infection.

Our platform provides a technical infrastructure that can simultaneously support telemedicine programmes for different chronic diseases and patients with different risk levels. Indeed, platforms like the Virtual Hospital are destined to play a major role in facilitating new strategies for chronic care management, for example, by means of out-of-hospital follow-up, patient empowerment and care coordination, thereby improving patient care without an associated rise in costs. Several chronic diseases such as diabetes, congestive heart failure or chronic obstructive pulmonary disease have all used telemedicine with similar findings [6]–[25].

The world's population is aging. As we age, the incidence and prevalence of chronic illness continue to rise, and elderly patients typically have multiple chronic diseases. The HIV-infected population follows this same trend, but the fact that the rate of new HIV infections has remained stable since 1995 [54] means the effect is worsened by the growing number of HIV-infected patients. The Hospital Clinic in Barcelona receives about 250 new HIV-infected patients each year. This increase, coupled with the drastic decrease in mortality due to current antiretroviral treatments, produces a significant annual increase in the care burden (around 10%, with an annual increase of 1000 new appointments in the Day Hospital). Given the current situation facing not only patients (who are likely to remain clinically stable for years) but also health providers, it is important to consider the enormous potential of the internet. Here the Virtual Hospital has been shown to be a feasible and safe tool for providing multidisciplinary home care to chronic HIV patients.

Although our study did not set out to measure the economics of study arms, to assist the reader's understanding of the importance of the work to health care providers and to society as a whole in implementing the new system, we have made a qualitative statement about whether, for example, cost savings could be expected with the new system. The population of this study for clinical monitoring of their infection, regardless of belonging to a study arm or another, required to perform a blood test, a medical visit and eventually, antiretroviral treatment.

Regarding costs of blood tests, both direct costs (unitary price of a standard blood analysis, a CD4 test and an HIV-1 RNA test) and indirect costs (transport costs and time invested to reach the laboratory, productivity cost, absenteeism, quality of life and psychosocial status) should be the same for both study arms, because this study contemplated to perform the blood tests on the same site.

Costs of medical consultations for the health system, as the estimated unit cost for an outpatient visit with an HIV specialist care provider would not be modified in both types of cares. However, indirect costs regarding the time that professionals spend on each visit, the resources on space, infrastructure and personnel needed for a consultation presumably would be lower in the telemedicine arm and higher the communication effectiveness between patients and professionals. For example, the median time of a consultation via telemedicine is usually 5 minutes, compared with 20 minutes of standard visits; virtual consultation can be made at any office with a computer connected to the Internet (it is not necessary a day care hospital unit investing large areas, technical infrastructure and staffing complex), the median time to response to a consultation in the virtual branch is usually 24 hours, thus minimizing possible deleterious effects that any delay in communication could cause on clinical management. In parallel the costs of medical visits for patients, both direct costs (transportation to hospital) and indirect costs would be diminished in the telematic care among others by reducing travel time or waiting time for visits and medications (the median Hvirtual waiting time consultation is about 10 minutes compared with 60–90 minutes of waiting time at standard care).

The direct costs of antiretroviral medicaments should also be the same in both cares, although indirect costs to obtain antiretrovirals in the virtual arm should be lower than in the standard care. In the standard care arm, the patient must go to the hospital pharmacy to pick up the medication, which would include additional costs in transportation and working-time losses as previously mentioned. In the virtual arm, either because the medication arrives directly to patient's home by courier or because it is the patient who chooses the best time to pick up medication when receives virtual antiretroviral prescription, indirect costs would be lower.

Then to summarize, out an initial software development investment, the Virtual Hospital cost per patient/year was 120 euros (or 70 euros without posting service cost medication delivery to patient home). Taking into account, the mentioned advantages that implementing this new model would provide in terms of direct and indirect costs of medical consultations and antiretroviral prescriptions for the health system and for patients, we believe that Virtual Hospital could be moreover cost-effective.

The study shows that Virtual Hospital constitutes a feasible, fairly satisfactory, safe and a low cost tool for the clinical care of stable HIV-infected patients and it has no deleterious effect on HIV clinical parameters and health services utilization.

Telemedicine should be considered an appropriate support service for the entire management of chronic HIV infection, is likely to prove extremely useful in settings with poor access to the health system.

## Supporting Information

Protocol S1. Trial Protocol.
(0.69 MB DOC)

Checklist S1. Consort Checklist.
(0.24 MB DOC)
