# Supplementary material for: A New Multidisciplinary Home Care Telemedicine System to Monitor Stable Chronic Human Immunodeficiency Virus-Infected Patients: A Randomized Study
Source: PLoS One. 2011 Jan 21;6(1):e14515. doi: 10.1371/journal.pone.0014515 (PMC3024968; doi:10.1371/journal.pone.0014515)
Supplement: Protocol S1. — Trial Protocol. (0.69 MB DOC) [file pone.0014515.s001.doc]

**Estudio piloto de la viabilidad de un Servicio de Telemedicina para enfermos infectados por el VIH estables, en estadío crónico de su enfermedad en el Hospital Clínic de Barcelona**

**ÍNDICE**

PROMOTORES E INVESTIGADORES DEL PROYECTO

[1. Resumen del Proyecto 6](#__RefHeading___Toc9769532)

[2. Introducción 9](#__RefHeading___Toc9769533)

[2.1. Antecedentes y estado actual de los conocimientos científico-técnicos 9](#__RefHeading___Toc9769534)

[2.2. Experiencia previa del equipo de investigación 10](#__RefHeading___Toc9769535)

[2.3. Justificación de los principios metodológicos del proyecto 13](#__RefHeading___Toc9769536)

[2.3.1. Perfil de los pacientes incluidos 13](#__RefHeading___Toc9769537)

[2.3.2. Control de los pacientes 13](#__RefHeading___Toc9769538)

[2.3.3. Tecnología 13](#__RefHeading___Toc9769539)

[3. Hipótesis del proyecto 15](#__RefHeading___Toc9769540)

[4. Objetivos del proyecto 16](#__RefHeading___Toc9769541)

[5. Metodología y plan de trabajo 17](#__RefHeading___Toc9769542)

[5.1. Criterios de eligibilidad 17](#__RefHeading___Toc9769543)

[5.1.1. Criterios de inclusión 17](#__RefHeading___Toc9769544)

[5.1.2. Criterios de exclusión 17](#__RefHeading___Toc9769545)

[5.2. Descripción del proyecto 18](#__RefHeading___Toc9769546)

[5.2.1. Descripción del sistema de telemedicina 18](#__RefHeading___Toc9769547)

[5.2.2. Desarrollo y arquitectura del sistema de telemedicina 19](#__RefHeading___Toc9769548)

[5.2.3. Evaluación del sistema en el Hospital Clínic 20](#__RefHeading___Toc9769549)

[5.3. Metodología de desarrollo 22](#__RefHeading___Toc9769550)

[5.4. Plan de trabajo 23](#__RefHeading___Toc9769551)

[6. Evaluación del proyecto 24](#__RefHeading___Toc9769552)

[6.1. Metodología de evaluación 24](#__RefHeading___Toc9769553)

[6.1.1. Evaluación técnica 24](#__RefHeading___Toc9769554)

[6.1.2. Evaluación clínica 25](#__RefHeading___Toc9769555)

[6.1.3. Evaluación económica y de organización 25](#__RefHeading___Toc9769556)

[7. Aparición de acontecimientos adversos 26](#__RefHeading___Toc9769557)

[7.1. Información mínima a especificar 26](#__RefHeading___Toc9769558)

[7.2. Criterios de imputabilidad 26](#__RefHeading___Toc9769559)

[7.3. Procedimientos para la notificación inmediata de los acontecimientos adversos graves o inesperados 27](#__RefHeading___Toc9769560)

[8. Incumplimientos del protocolo 28](#__RefHeading___Toc9769561)

[8.1. Abandono del tratamiento, negativa a seguir 28](#__RefHeading___Toc9769562)

[8.2. Aparición de un embarazo 28](#__RefHeading___Toc9769563)

[9. Medidas de seguridad 29](#__RefHeading___Toc9769564)

[9.1. Ética y confidencialidad de los datos. 29](#__RefHeading___Toc9769565)

[9.1.1. Aprobación del estudio por el Comité de Etica y de Investigación Clínica 29](#__RefHeading___Toc9769566)

[9.1.2. Confidencialidad de los datos. 29](#__RefHeading___Toc9769567)

[10. Infraestructura 30](#__RefHeading___Toc9769568)

[11. Presupuesto 31](#__RefHeading___Toc9769569)

ANEXO I: Indice de Karnofsky

ANEXO II: Nota informativa para los pacientes incluidos en el estudio

ANEXO III: Formulario de consentimiento informado

ANEXO IV: Hoja de notificación de acontecimientos adversos

**INVESTIGADORES DEL PROYECTO**

**PROMOTORES**

Prof. José Mª Gatell Artigas

Servicio de Enfermedades Infecciosas

Hospital Clinic i Provincial

Villarroel, 170

08036 - Barcelona

Tel. 93-2275430

Fax 93-4514438

e-mail: gatell@medicina.ub.es

Prof. Francisco del Pozo Guerrero

Grupo de Bioingeniería y Telemedicina

ETSI Telecomunicación

Universidad Politécnica de Madrid

Ciudad Universitaria s/n

28040 Madrid (SPAIN)

Tel: +34 - 915495700 ext. 332

Fax: +34 – 913366828

e-mail: fpozo@gbt.tfo.upm.es

**INVESTIGADORES PRINCIPALES**

Dr. Felipe García Alcaide

Servicio de Enfermedades Infecciosas

Hospital Clinic i Provincial

Villarroel, 170

08036 - Barcelona

Tel. 93-2275586

Fax: 93-4514438

e-mail: fgarcia@medicina.ub.es

Prof. Enrique J. Gómez Aguilera

Grupo de Bioingeniería y Telemedicina

ETSI Telecomunicación

Universidad Politécnica de Madrid

Ciudad Universitaria s/n

28040 Madrid (SPAIN)

Tel: +34 - 915495700 ext. 332

Fax: +34 - 913366828

e-mail: egomez@gbt.tfo.upm.es

**MONITOR DEL ESTUDIO**

Dra. Anna Cruceta

Servicio de Enfermedades Infecciosas

Hospital Clinic i Provincial

Villarroel, 170

08036 - Barcelona

Tel. 93- 4515424. Fax 4514438

**INVESTIGADORES ASOCIADOS**

José Mª Gatell

Josep M Miró

Josep Mallolas

Esteban Martínez

Begoña Ramírez

Laura Zamora

Teresa Mejías

Jordi Blanch*

Menchu Lanaspa**

Carlos Codina***

Instituto Clínic d’Infeccions i Immunologia, *Psiquiatría, **Trabajo Social, ***Farmacia

**Hospital Clínic** Villarroel, 170. 08036-Barcelona. Tel. 93-2275430

Francisco del Pozo

Mª Elena Hernando

César Cáceres

Ángel García

Paula de Toledo

Santiago Rodríguez

**Grupo de Bioingeniería y Telemedicina** (Universidad Politécnica de Madrid)

Ciudad Universitaria s/n, 28040 – Madrid. Tel. 91-5495700 ext. 332

Xavier Badía

**Health Outcomes Research Europe**

Plató 6 1º 5ª 08021 Barcelona Teléfono: 93 2093257 Fax: 93 2412710

# Resumen del Proyecto

**Objetivo**

Este proyecto tiene por objetivo la definición, desarrollo, instalación en rutina clínica y evaluación de un servicio de telemedicina que asocie a la atención estándar un seguimiento “virtual” para el cuidado de enfermos infectados por el VIH estables, en estadío crónico de su enfermedad y estudiar si mejora la calidad asistencial y el gasto por enfermo respecto al control habitual sin servicio de telemedicina que realizan los enfermos.

**Diseño**

Como tipo de experimento para realizar la evaluación del sistema se propone un estudio piloto randomizado cruzado abierto prospectivo.

**Lugar**

Este sistema de telemedicina será integrado y evaluado en el Servicio de Enfermedades Infecciosas del Hospital Clínic de Barcelona.

**Criterios de inclusión**

- Infección por VIH
- En tratamiento con TARGA al menos durante 3 meses
- Consentimiento informado por escrito
- Disponer de domicilio con conexión telefónica
- Indice de Karnofsky superior al 80% (Anexo I)

**Criterios de exclusión**

- Pacientes VIH en fracaso terapéutico (linfocitos CD4 < 200 ml o carga viral > 20.000 copias/ml)
- Pacientes que no se encuentren en un estadío estable de su enfermedad (primoinfectados, con infecciones oportunistas)
- Edad < 18 años
- Previsión de cumplimiento incorrecto del tratamiento o vigilancia de difícil realización
- Mujeres embarazadas o lactantes o con previsión de quedar embarazadas
- Consentimiento denegado

**Intervención**

Según información disponible, aproximadamente un 25% de los pacientes VIH consultan Internet regularmente para informarse sobre su enfermedad. En el Hospital Clínic se controlan unos 3000 pacientes, por lo que se podrían beneficiar de este programa unos 750 pacientes. Al ser un estudio piloto se seleccionarían 100 pacientes con acceso telefónico en su domicilio.

Los 100 pacientes serían randomizados:

- Rama A: recibir control normal + Servicio de Telemedicina (n=50).
- Rama B: recibir control habitual en hospital de día de VIH (n=50).

Los pacientes a los que se añada el Servicio de Telemedicina al control normal realizarán dos visitas reales al año, en vez de las 4 visitas que se realizan habitualmente. Además se realizarán otras 2 visitas virtuales para control de analítica y medicación.

Es importante tener en cuenta que los usuarios del sistema no serán únicamente los pacientes, sino que se incluye también al resto de personal que trabaje en el proyecto. Se constituirá por tanto un equipo de profesionales formado por: una enfermera, un médico, un psiquiatra o psicólogo, un trabajador social, un farmacéutico, un experto en calidad de vida y un administrador.

**Funcionalidades del sistema de telemedicina**

La estructura de sistema sería la construcción de un sistema web avanzado que proporcionará los siguientes servicios a los usuarios:

Para las personas que están infectadas por el VIH:

- Consultas virtuales
- Gestión de la medicación
- Gestión de la información
- Gestión de la comunidad virtual de pacientes

Para los profesionales que atienden a personas infectadas por VIH dispondrían, además de los anteriores, de los siguientes servicios especiales:

- Gestión de la comunidad virtual de profesionales
- Comunicación continua entre los profesionales del equipo sanitario
- Evaluación de los efectos secundarios y cumplimiento de la medicación
- Evaluación de aspectos psicológicos de los pacientes
- Evaluación de calidad de vida de los pacientes
- Evaluación de gastos, farmacoeconomía,...

**Criterios de valoración**

Se realizarían las siguientes evaluaciones a los dos grupos de pacientes:

1. Calidad asistencial

- Control de la infección por VIH, medido por respuesta virológica e inmunológica al tratamiento antirretroviral, fracasos de la medicación, desarrollo de resistencias, y en su caso infecciones oportunistas y/o muerte
- medición de adherencia a la medicación y sus efectos secundarios antes y después de comienzo del estudio
- calidad de vida y aspectos psicosociales del paciente antes y durante el estudio

2. Gasto por enfermo

- coste en medicamentos antes y después del comienzo del estudio
- parámetros farmacoeconómicos
- coste total del control del paciente en un año

3. Aspectos técnicos

La evaluación se puede plantear en dos fases: una primera experiencia piloto con diez pacientes por rama, que se evaluará tras seis meses de seguimiento, y una evaluación posterior alcanzando un total de 100 pacientes (50 en cada rama) durante 2 años.

**Duración del estudio**

El estudio tendría que durar un año para su planteamiento técnico y dos años a partir de la primera visita al primer paciente. Habrá primero una fase piloto con 10 pacientes por rama que se evaluará tras 6 meses de seguimiento, posteriormente se seguirá el resto del estudio.

**Ética y confidencialidad de los datos**

Habrá un comité de seguridad independiente que controlará en todo momento el proyecto.

# Introducción

## Antecedentes y estado actual de los conocimientos científico-técnicos

Gracias a la disponibilidad de los nuevos tratamientos antirretrovirales, la infección por VIH ha pasado a ser una enfermedad crónica, aunque es imposible su curación. Actualmente, los pacientes controlados correctamente pueden tener una vida normal con un control médico y, en muchos casos, una medicación adecuada. Sin embargo, incluso en los pacientes estables el manejo es complejo, precisándose un personal altamente cualificado. La complejidad viene derivada de diversos factores:

- La propia infección: es una infección que requiere un control cercano con 4-6 visitas al año. En estas visitas se hace un control clínico y analítico (virológico, inmunitario, general).
- La medicación: se requiere un alto grado de adherencia (mayor al 95%) a una medicación compleja, con frecuentes efectos secundarios y problemas de tolerancia.
- Problemas psicológicos y sociales: es una infección que significa un problema importante para el paciente desde el punto de vista de sus relaciones personales (vida sexual, manejo diario, trabajo) y conlleva una sobrecarga psicológica importante.

Todas estas características inducen a pensar que una mayor participación de los pacientes en su enfermedad signifique un mejor control y una mejora en la calidad de vida. En este campo la educación sanitaria, el acceso fácil de los pacientes a la consulta de dudas diarias, la participación del paciente en la toma de decisiones conllevará con mucha probabilidad un mejor control del paciente.

Actualmente, la telemedicina puede ayudar a un mejor control de los pacientes. La telemedicina es una herramienta que debería servir no para sustituir a la relación clásica médico-paciente (como se plantea en algunos sitios de hospitales únicamente virtuales), sino para mejorar esta relación, haciendo que el paciente participe en su propio control (como ya se hace con éxito en otras enfermedades crónicas como la diabetes). Esta mayor implicación del paciente en su enfermedad requiere una disponibilidad de información y autogestión que sólo la telemedicina nos puede ofrecer.

Muy probablemente esta implicación conlleve una mayor satisfacción del paciente, pero además debe conllevar un mejor control del mismo, asegurando las tomas de medicación, minimizando los efectos secundarios y, por último, implicando un ahorro económico importante tanto en gasto farmacéutico como de control del paciente.

Las tecnologías que se conocen y se aplican en esta línea de investigación son el desarrollo de sistemas de monitorización ambulatoria, protocolos de telemedicina, estaciones de trabajo médicas, integración de aplicaciones multi-plataforma, trabajo cooperativo, servicios de mensajería, análisis de datos, gestión inteligente de la información y gestión del conocimiento y sistemas de ayuda a la decisión.

## Experiencia previa del equipo de investigación

Con el fin de abarcar todos los ámbitos del proyecto, se ha constituido un equipo de investigación multidisciplinar que colabora estrechamente en todas las etapas de desarrollo y evaluación. Este equipo está compuesto por los siguientes grupos de investigación:

Servicio de Enfermedades Infecciosas del Hospital Clínic de Barcelona, dirigido por el Prof. José M Gatell. Este servicio atiende en la actualidad unos 3.000 pacientes infectados por el VIH. Es el centro europeo que atiende a un mayor número de enfermos con SIDA, con la excepción del Hospital Pitié-Salpetrière de Paris. Además, este equipo de investigación es uno de los grupos líderes en investigación básica y aplicada en VIH/SIDA, y así mismo, con una larga tradición en la aplicación de tecnologías innovadoras en evaluación de la calidad asistencial y promoción de la comunicación entre los diferentes niveles de la organización sanitaria y el paciente con VIH/SIDA. Para la evaluación y puesta en marcha del proyecto se contará asimismo con un experto en calidad de vida.

Durante los últimos años se han realizado aportaciones y publicaciones originales que superan los 350 puntos de factor impacto. Concretamente en el campo del tratamiento antirretroviral se ha participado en todos los estudios internacionales que han introducido nuevos medicamentos en nuestro país. Varios de estos estudios han sido diseñados en nuestro centro. El Dr. Gatell ha formado parte de los comités internacionales de coordinación entre otros del estudio BW020 publicado en el New England Journal of Medicine que estableció por primera vez las ventajas de tratar pacientes asintomáticos en fases muy precoces de la enfermedad y del estudio AVANTI que por primera vez ha introducido en nuestro país combinaciones triples y cuadruples de antirretrovirales incluyendo inhibidores de las proteasas.

A nivel europeo (UE) el Dr Gatell fue investigador principal de una Acción Concertada para el Estudio de la Tuberculosis y es miembro del Steering Committee”, así de otras dos (proyectos del programa BIOMED-2): ENTA (European Network for Treatment of AIDS) y EUROAIDS cuya finalidad es el diseño y coordinación de estudios europeos y la recogida prospectiva de datos epidemiológicos del SIDA en Europa para evaluar las variaciones a lo largo del tiempo. El grupo se financia con aportaciones privadas, ayudas FISss (ver apartado correspondiente), ayudas de la Unión Europea y ayudas de la MICYT.

En cuanto a la experiencia en Internet, los Dres Gatell y Felipe García han desarrollado un proyecto educativo desde 1998 de sida en internet ([www.prous.com/ttmsida](http://www.prous.com/ttmsida)) en este proyecto además se pueden realizar consultas directamente a expertos un servicio que ha atendido a más de 1000 consultas hasta la actualidad. Este proyecto está reconocido y apoyado por la Sociedad Española de Enfermedades Infecciosas y Microbiología Clínica, la Sociedad Española de Quimioterapia y la Sociedad Española Interdisciplinaria del SIDA. Asimismo, es un Programa acreditado por la Comisión de Formación Continuada del Sistema Nacional de Salud y por el Consell Català de Formació Mèdica Continuada.

Servicio de Farmacia del Hospital Clínic de Barcelona. Este Servicio es pionero en atención a pacientes infectados por VIH. Se ha prestado especial atención a la información sobre los medicamentos para los pacientes (con un servicio informatizado), atención farmacéutica individualizada, estudios sobre adherencia,...

Grupo de Bioingeniería y Telemedicina (Dpto. Tecnología Fotónica de la ETSI Telecomunicación) de la Universidad Politécnica de Madrid, dirigido por el Prof. Francisco del Pozo. El GBT es el mayor centro de investigación español en telemedicina y mantiene colaboraciones estables con el sector privado y público en diversas áreas de I+D. El centro está especializado en el diseño, desarrollo y evaluación de tecnologías, servicios y aplicaciones de telemedicina en áreas como: 1) el cuidado domiciliario; 2) servicios de información sanitaria en Web; 3) sistemas multimedia y multimodales para personas con necesidades especiales; 4) teleradiología avanzada y diagnóstico cooperativo en imagen médica; 5) telecuidado de pacientes diabéticos; 6) telemedicina en países en vías de desarrollo; 7) sistemas y servicios para gestión de emergencias; y 8) realidad virtual en medicina. También es importante destacar su experiencia previa en el modelado, definición, desarrollo y evaluación de servicios de telemedicina, y su trabajo, durante los quince años anteriores en diversos proyectos de investigación nacionales y europeos, en el campo de la monitorización ambulatoria y telecuidado de pacientes crónicos, así como en sistemas de ayuda al diagnóstico, monitorización y planificación de terapias (principalmente en diabetes y VIH/SIDA).

Para ilustrar con ejemplos la experiencia de este grupo de investigación, citamos a continuación algunos de los proyectos de investigación realizados por el Grupo de Bioingeniería y Telemedicina en el campo del cuidado de pacientes crónicos:

**Proyecto SEAHORSE II**. (HC-4019. 1998-2000). “SEAHORSE II: Support, Empowerment and Awareness for HIV/AIDS: the On-line Research and Self-help Exchange”. Este proyecto europeo tenía por objetivo la creación de un servicio de información web sobre VIH/SIDA revisado y validado por expertos, así como servicios de auto-seguimiento de pacientes VIH/SIDA. El equipo investigador desarrolló el módulo de auto-seguimiento a través de Internet de pacientes seropositivos.

**Proyecto Chronic**. (IST-1999-12158. 2000-2002). El objetivo de este proyecto europeo es desarrollar un nuevo modelo europeo para el cuidado de pacientes crónicos basado en un entorno integrado TIC que implementa un “homehub” domiciliario para diversas patologías y en concreto para la enfermedad obstructiva pulmonar crónica (EPOC). El equipo investigador es responsable de la definición de los modelos de telecuidado y de la provisión de las aplicaciones de telemedicina que serán probadas en varios sitios piloto europeos, siendo uno de ellos el Hospital Clínic de Barcelona.

**Proyecto Healthmate**. (IST-2000-26154. 2001-2003). El proyecto europeo pretende el desarrollo de un sistema portátil para servicios de telecuidado y teleconsulta que incorpore tecnologías de comunicaciones inalámbricas y protocolos entre estos sistemas y los proveedores de servicios sanitarios.

**Proyecto HERO**. (IST-2000-26724. 2001-2003). “HERO: Health Promotion and Educational Support for the Rehabilitation of Offenders”. Este proyecto europeo pretende desarrollar un sistema de promoción de salud y educación en prisiones, para lo cual se centra en los presos preventivos y en los que saldrán a corto plazo de prisión.

**Proyecto T-IDDM**. (HC-1047. 1995-1999). "T-IDDM: Telematic management of insulin dependent diabetic mellitus”. Este proyecto europeo tenía por objetivo la definición de un sistema de telemedicina basado en un servidor Web que soportara un sistema basado en conocimiento para la definición de estrategias terapéuticas de ajuste de insulina.

**Proyecto DIABTel**. (CICYT-SAF98-0170. 1998-2000). “DIABTel: Telemedicina en el cuidado de la Diabetes Mellitus”. Este proyecto nacional de investigación tenía por objetivo definir, desarrollar, instalar y evaluar un servicio de telemedicina para el cuidado de la diabetes. El proyecto consiguió desarrollar, instalar y evaluar el sistema DIABTel en rutina clínica en el Hospital Sant Pau de Barcelona, demostrándose las hipótesis de partida del proyecto como la posibilidad de realizar nuevos protocolos de telemedicina en los que se acortaran los tiempos de respuesta en la valoración del estado metabólico del paciente por parte del médico y la provisión de un servicio de “telecuidado” desde el hospital al entorno de vida del diabético, complementado por el servicio de “autonomía supervisada”.

**Proyecto M2DM**. (IST-1999-10315. 2000-2002). El proyecto europeo M²DM propone un nuevo sistema multi-acceso de telecuidado de pacientes diabéticos para la provisión de un servicio universal, ubícuo y personalizado de comunicación, compartición y acceso a información para profesionales y pacientes integrando un conjunto de soluciones TIC (medidores de glucosa, Web-TV y CTI), adaptado a los conocimientos tecnológicos, localización física o responsabilidad en el equipo de cuidado del usuario.

**Proyecto Mobcare**. (HC-4014. 1998-1999). El objetivo de este proyecto europeo era el diseño de un sistema de cuidado domiciliario basado en un sistema de comunicaciones móviles. El trabajo del equipo investigador en el proyecto consistió en el diseño y desarrollo de un sistema de cuidado de pacientes con complicaciones respiratorias.

## Justificación de los principios metodológicos del proyecto

### Perfil de los pacientes incluidos

Los criterios escogidos son que sean pacientes que se encuentren en una situación estable, dado que la rutina asistencial de estos pacientes facilitaría la utilización de un sistema informático y al ser un estudio piloto con pacientes homogéneos se podrían valorar mejor los resultados.

### Control de los pacientes

Los pacientes pasarán a controlarse con visitas reales 2 veces al año en vez de las 4 veces que realizan actualmente. Además se realizarán otras 2 visitas virtuales para control de analítica y medicación. El control de estos pacientes se llevará a cabo por:

- Una enfermera con una hora diaria de visita real y otra hora de visita virtual (total 2 horas al día). Su labor sería los cuidados tradicionales de enfermería en la hora real. En la hora virtual sería una labor de educación, sanitaria, información y asesoramiento.
- Un médico en una consulta real de una hora y virtual de otra hora diaria (total 2 horas al día). La visita real sería el cuidado médico tradicional, mientras que el virtual sería atención de consultas médicas concretas sobre efectos secundarios, explicación de analíticas,...
- También se contaría con un psiquiatra/psicólogo doce horas a la semana.
- Un trabajador social con dos horas de atención real y una hora virtual (total 3 horas a la semana).
- Acceso al sistema para control y evaluación por parte de un farmacéutico, un experto en calidad de vida y un administrador del proyecto.

### Tecnología

La elección tecnológica del proyecto viene determinada por largos años de experiencia del equipo investigador en el campo de la telemedicina. En el caso del cuidado de pacientes crónicos, lo que se pretende es reforzar la comunicación profesional-paciente y profesional-profesional. Para ello se dispone de las tecnologías Internet muy presentes hoy en día en la sociedad de la información.

Por tanto la plataforma de desarrollo del sistema elegida es Internet, desde donde se pretende ofrecer diversos servicios web principalmente en los siguientes aspectos:

- Comunicación Profesional-Paciente: para potenciar esta comunicación se utilizará un sistema de videoconferencia que mejorará enormemente la comunicación habitual de sistemas de consulta virtual que es a través del intercambio de correos electrónicos. La consulta virtual por videoconferencia ofrece una mayor cercanía en esa relación básica con el paciente para los profesionales sanitarios.
- Comunicación entre los profesionales: para mejorar la comunicación en el cuidado compartido de un paciente crónico, el sistema ofrece una comunidad virtual en la que los profesionales pueden intercambiarse información para mejorar su coordinación y trabajo en equipo.

# Hipótesis del proyecto

Los nuevos paradigmas de cuidado compartido de pacientes crónicos basados en el uso adecuado y optimizado de los avances tecnológicos se fundamentan en la creación de equipos donde el paciente sea un integrante más, con responsabilidad en su propia enfermedad. Una mayor comunicación y de mejor calidad entre los miembros de estos equipos junto con el acceso a servicios de telemedicina (como las consultas virtuales) conllevarían:

- una mayor satisfacción del paciente
- un mejor control de la enfermedad
- mejoraría la adherencia a la medicación
- minimizaría los efectos secundarios
- un ahorro económico importante tanto en gasto farmacéutico como de control del paciente

# Objetivos del proyecto

Este proyecto tiene por objetivo principal la definición, desarrollo, instalación en rutina clínica y evaluación de un servicio de telemedicina que combine la atención “real” con la “virtual” para el cuidado de enfermos infectados por el VIH estables, en estadío crónico de su enfermedad. Este sistema de telemedicina será integrado y evaluado en el Servicio de Enfermedades Infecciosas del Hospital Clínic de Barcelona.

Los objetivos concretos del proyecto son:

- La definición de un modelo de cuidado de pacientes infectados por el VIH basado en telemedicina.
- La definición y desarrollo de un sistema de telemedicina basado principalmente en tecnologías Internet.
- La integración de un sistema de análisis inteligente de datos de monitorización en el sistema de telemedicina.
- La definición de un plan estratégico de implantación del sistema de telemedicina en rutina clínica.
- La evaluación del sistema en el hospital considerando los aspectos técnicos, clínicos y económico-organizativos.

# Metodología y plan de trabajo

Como tipo de experimento para realizar la evaluación del sistema se propone un **estudio piloto randomizado cruzado abierto prospectivo**, dividiendo a los pacientes en dos ramas:

- Rama A: reciben el control habitual junto con la atención con el sistema de telemedicina.
- Rama B: reciben el control habitual en el hospital de día de VIH.

Los pacientes que utilicen el sistema de telemedicina realizarán dos visitas reales al año, en vez de las 4 visitas que se realizan habitualmente. Además se realizarán otras 2 visitas virtuales para control de analítica y medicación.

## Criterios de eligibilidad

### Criterios de inclusión

- Infección por VIH
- En tratamiento con TARGA al menos durante 3 meses
- Consentimiento informado por escrito
- Disponer de domicilio con conexión telefónica
- Índice de Karnofsky superior al 80% (Anexo I)

### Criterios de exclusión

- Pacientes VIH en fracaso terapéutico (linfocitos CD4 < 200 ml o carga viral > 20.000 copias/ml)
- Pacientes que no se encuentren en un estadío estable de su enfermedad (primoinfectados, con infecciones oportunistas)
- Edad < 18 años
- Previsión de cumplimiento incorrecto del tratamiento o vigilancia de difícil realización
- Mujeres embarazadas o lactantes o con previsión de quedar embarazadas
- Consentimiento denegado

## Descripción del proyecto

A continuación se describe el proyecto, tanto en sus aspectos funcionales como en su desarrollo técnico y posterior evaluación clínica.

### Descripción del sistema de telemedicina

El sistema de telemedicina (Hospital VIHrtual) proporcionaría los siguientes servicios a los distintos usuarios a través de Internet:

- Para las personas que están infectadas por el VIH:
- Consultas virtuales: enfermería, médicas, el equipo de atención psicológica, equipo de atención social, farmacéutico. Serán de 3 tipos: visita virtual concertada (mediante videoconferencia), consulta virtual (en el tiempo de visita virtual del personal sanitario, mediante chat), consulta virtual esporádica (mediante mensaje electrónico).
- Gestión de la medicación: solicitud de medicamentos por parte del equipo médico a Farmacia y envío a domicilio. Notificación de cambios en la terapia. Se dispondrá de herramientas de monitorización del tratamiento (cumplimiento, efectos secundarios,...).
- Gestión de la información: disponibilidad de acceso ilimitado a una información elaborada para pacientes sobre la enfermedad, medicamentos, efectos secundarios, posibilidades de futuro, ensayos clínicos, innovaciones, acceso a recursos de la zona, noticias...
- Gestión de la comunidad virtual: participación del paciente en una comunidad virtual con chat para compartir experiencias con otros enfermos. Asimismo, posibilidad de recibir y participar en charlas informativas periódicas con el equipo sanitario.
- Para los profesionales que atienden a las personas infectadas por VIH, dispondrían de los mismos servicios que los anteriores, con las siguientes distinciones:
- Gestión de la comunidad virtual: participación del profesional en una comunidad virtual con chat para realizar sesiones clínicas virtuales.
- Comunicación continua entre el equipo sanitario mediante plataformas multiacceso (web, PDA, GSM,...).
- Ayuda para el control de los efectos secundarios y del cumplimiento de la medicación.
- Análisis inteligente de los datos de monitorización: este análisis se puede dividir en dos etapas: 1) un estudio previo retrospectivo de los innumerables datos almacenados a lo largo de los años en la base de datos del Servicio de Enfermedades Infecciosas, mediante el uso de técnicas de minería de datos para extraer características tanto de la enfermedad y sus tratamientos como de los pacientes y sus perfiles; y 2) el uso de los datos extraídos en el estudio anterior para mejorar tanto la asistencia a los pacientes actuales como la investigación en el empleo de las distintas terapias existentes.
- Evaluación de aspectos psicológicos de los pacientes mediante cuestionarios web.
- Evaluación de calidad de vida de los pacientes mediante cuestionarios web.
- Evaluación de gastos, farmacoeconomía,...

### Desarrollo y arquitectura del sistema de telemedicina

Se desarrollará un sistema de telemedicina que integre las funcionalidades anteriormente expuestas. Este desarrollo se puede dividir en los distintos bloques de tareas:

- Tareas de seguridad
- Tareas de videoconferencia
- Tareas de gestión de usuarios
- Tareas de evaluación
- Tareas de gestión del intercambio de datos clínicos
- Tareas de creación y gestión de comunidades virtuales
- Tareas de gestión del multiacceso
- Tareas de gestión de tratamientos
- Tareas de análisis inteligente de datos
- Tareas de gestión de la información
- Tareas de diseño de la interfaz de usuario

La arquitectura sobre la que se desarrollará el sistema de telemedicina es la representada en la figura 1, en la que se observa cómo se accede a la zona segura donde están el servidor y la base de datos del Hospital VIHrtual. También se muestra cómo los profesionales pueden acceder bien desde una red de área local (LAN) en el hospital con el ordenador o la agenda electrónica (PDA), bien desde el exterior a través de Internet (como es el caso del experto en calidad de vida); mientras que los pacientes pueden acceder desde sus domicilios a través de Internet con su ordenador o terminal webTV.

Figura 1. Arquitectura del sistema de telemedicina

### Evaluación del sistema en el Hospital Clínic

Para la evaluación del sistema de telemedicina, se contará con dos experimentos de 20 y 100 pacientes, de seis meses y dos años de duración respectivamente. Estos estudios serán llevados a cabo por el grupo de investigación del Hospital Clínic de Barcelona formado por:

***Equipo médico***

El tratamiento clínico será el mismo para los pacientes con y sin sistema, disponiéndose en ambos grupos de:

- Historia Clínica Completa del paciente, que incluirá edad, sexo, grupo de riesgo, fecha aproximada de contagio, si hubo primoinfección sintomática, otros antecedentes patológicos (enfermedades de transmisión sexual, hepatitis crónica, herpes zoster) y vacunaciones recibidas.
- Análisis biológicos (cada 3 meses): Hematológicos (hemoglobina, hematocrito, leucocitos totales, polinucleares neutrófilos, plaquetas), Bioquímicos (creatinina, transaminasas (SGOT o ASAT, SGPT o ALAT), fosfatasa alcalina, gammaglutamiltranspeptidasa, bilirrubina, colesterol, triglicéridos, glucemia, amilasas, lipasas), Inmunológicos (subpoblaciones linfocitarias CD4 y CD8, se consignarán los valores absolutos y los porcentajes) y Carga viral (número de copias de ARN viral).
- Medicación: se mantendrá la medicación que tome el paciente.

La principal diferencia entre utilizar el sistema de telemedicina y no utilizarlo estriba en las consultas ya que, usando el Hospital VIHrtual, estas podrán ser virtuales.

El equipo médico dispondrá para atender a los pacientes del estudio de dos horas al día distribuidas de la siguiente forma:

- Una consulta real de una hora diaria, en la que se realiza el seguimiento y cuidado médico tradicional.
- Una consulta virtual de otra hora diaria, que podrá dividirse a su vez en:
- Consulta virtual programada: se trata de la consulta real, pero a través de videoconferencia.
- Consulta virtual puntual: donde se atenderían dudas en tiempo real (mediante chat) sobre efectos secundarios, explicación de analíticas...
- Consulta virtual puntual a cualquier hora: Cualquier duda no urgente que tenga el paciente y que el equipo médico responderá en cuanto pueda por correo electrónico.

***Equipo de enfermería***

Este equipo cuenta, al igual que el médico, con dos horas al día para atender a los pacientes incluidos en el estudio, de la siguiente manera:

- Una hora diaria de consulta real, donde recibiría el trato habitual (información al paciente y a su entorno, prevención, control de la adhesión al tratamiento, valoración general del paciente, mejora de la calidad de vida del paciente facilitándole recursos, derivación a otros profesionales,...)
- Una hora diaria de consulta virtual, donde además de realizar las labores propias de la consulta real, resolvería dudas de los pacientes y les facilitaría información a través de Internet.

***Equipo de psiquiatría/psicología***

También se cuenta con un psiquiatra o psicólogo con doce horas de atención a la semana.

- Consultas reales, donde se realizaría la entrevista psiquiátrica inicial (en la que se realiza la anamnesis de trastornos psiquiátricos y la exploración psicopatológica) y las entrevistas de seguimiento terapéutico.
- Consultas virtuales donde se podrán hacer también entrevistas de seguimiento terapéutico, y además el paciente podrá plantear sus dudas y rellenar una serie de cuestionarios para la valoración psiquiátrica-psicológica (Cuestionario General de Salud (GHQ); Escala Hospitalaria de Ansiedad y Depresión (HADS); Lista de 90 síntomas psicopatológicos (SCL-90); Escala de adaptación psicosocial a la enfermedad (PAIS)).

***Equipo de atención social***

En este equipo, un trabajador social atenderá a los pacientes tres horas a la semana. Tanto la consulta real (dos horas/semana) como la virtual (una hora/semana) serán entrevistas de información, orientación, asesoramiento, apoyo y motivación al enfermo y/o familia.

***Equipo farmacéutico***

El Servicio de Farmacia atenderá las consultas de los pacientes sobre medicamentos, haciendo un seguimiento especial de las reacciones adversas y la adherencia. También supervisará el envío de los medicamentos, el recuento de la medicación sobrante y la información que se ofrece a los pacientes.

***Equipo experto en calidad de vida***

Debe comprobar si se dan diferencias en cuanto a la Calidad de Vida del paciente infectado por VIH dependiendo de la rama de control a la que pertenezca. El seguimiento se realiza a través de tres cuestionarios: Cuestionario de Calidad de vida genérico (EuroQol-5D), Cuestionario de Calidad de vida específico para paciente infectados de VIH (MOS-HIV) y una Escala de Satisfacción con la asistencia sanitaria. Durante el estudio habrá un especialista en Calidad de Vida con el que, ante cualquier duda, problema o consulta, tantos los investigadores como los pacientes puedan contactar.

***Un administrador del proyecto***

Se encargará de todos los aspectos relacionados con facturación, costes, pagos a proveedores o personal,...

## Metodología de desarrollo

La organización de las actividades del proyecto se realiza en módulos de trabajo (o paquetes de trabajo). Estos son los siguientes:

MÓDULO 1: Modelado, análisis de requisitos y plan estratégico

MÓDULO 2: Diseño y especificación del sistema de telemedicina

MÓDULO 3: Desarrollo del sistema “Hospital VIHrtual”

MÓDULO 4: Integración de módulos y verificación técnica

MÓDULO 5: Instalación e integración del sistema en el hospital

MÓDULO 6: Evaluación del sistema

MÓDULO 7: Explotación de resultados e impacto económico-social

MÓDULO 8: Coordinación y dirección científico-técnica

Cada módulo genera un resultado en forma de documento y/o prototipo que denominaremos “hito”. Existen por tanto ocho hitos a lo largo del proyecto, teniendo los hitos 4, 6 y 8 especial relevancia por lo que los destacamos también en el cuadro del Plan de Trabajo. Los hitos de cada módulo son:

HITO 1 – Modelo de sistema telemédico, análisis de requisitos de usuario y plan de implantación del sistema

HITO 2 – Especificaciones de diseño y funcionalidades del sistema

HITO 3 – Prototipos de los distintos módulos del sistema de telemedicina

HITO 4 – Prototipo 1 del sistema de telemedicina “Hospital VIHrtual 1”

HITO 5 – Guía de instalación y Manual de usuario

HITO 6 – Prototipo final del sistema de telemedicina “Hospital VIHrtual final”

HITO 7 – Explotación de resultados y análisis del impacto económico-social

HITO 8 – Memoria final del estudio

El prototipo en versión 1 se produce al final del correspondiente módulo de desarrollo (HITO 4) y el prototipo final (HITO 6) después de la evaluación y refinamiento de los sistemas al segundo año de proyecto (ver cuadro del Plan de Trabajo) para comenzar su evaluación, que finalizará con la Memoria final del proyecto (HITO 8).

## Plan de trabajo

La planificación temporal propone lograr un primer prototipo del sistema para el primer año del estudio (HITO 4). Este prototipo no incluiría algunas funcionalidades complejas, como el análisis inteligente de los datos de monitorización o las comunidades virtuales, ya que para estas tareas se prevén unos tiempos de desarrollo mayores. El sombreado claro de la figura indica los tiempos necesarios para rediseñar, incluir las nuevas especificaciones que vayan apareciendo e implementarlas en el sistema final (HITO 6) al finalizar el segundo año del proyecto.

|  |  |  |  | AÑO 1 | | | | | | | | | | | | AÑO 2 | | | | | | | | | | | | AÑO 3 | | | | | | | | | | | |
| --- | --- | --- | --- | --- | --- | --- | --- | --- | --- | --- | --- | --- | --- | --- | --- | --- | --- | --- | --- | --- | --- | --- | --- | --- | --- | --- | --- | --- | --- | --- | --- | --- | --- | --- | --- | --- | --- | --- | --- |
|  |  |  |  | 1 | 2 | 3 | 4 | 5 | 6 | 7 | 8 | 9 | 10 | 11 | 12 | 1 | 2 | 3 | 4 | 5 | 6 | 7 | 8 | 9 | 10 | 11 | 12 | 1 | 2 | 3 | 4 | 5 | 6 | 7 | 8 | 9 | 10 | 11 | 12 |
| 1: Modelado, análisis de requisitos y plan estratégico | | |  |  |  |  |  |  |  |  |  |  |  |  |  |  |  |  |  |  |  |  |  |  |  |  |  |  |  |  |  |  |  |  |  |  |  |  |  |
| 2: Diseño y especificación del sistema de telemedicina | | | |  |  |  |  |  |  |  |  |  |  |  |  |  |  |  |  |  |  |  |  |  |  |  |  |  |  |  |  |  |  |  |  |  |  |  |  |
| 3: Desarrollo del sistema “Hospital VIHrtual” | | | |  |  |  |  |  |  |  |  |  |  |  |  |  |  |  |  |  |  |  |  |  |  |  |  |  |  |  |  |  |  |  |  |  |  |  |  |
| 4: Integración de módulos y verificación técnica | | | |  |  |  |  |  |  |  |  |  |  |  |  |  |  |  |  |  |  |  |  |  |  |  |  |  |  |  |  |  |  |  |  |  |  |  |  |
| 5: Instalación e integración del sistema en el hospital | | | |  |  |  |  |  |  |  |  |  |  |  |  |  |  |  |  |  |  |  |  |  |  |  |  |  |  |  |  |  |  |  |  |  |  |  |  |
| 6: Evaluación del sistema | |  |  |  |  |  |  |  |  |  |  |  |  |  |  |  |  |  |  |  |  |  |  |  |  |  |  |  |  |  |  |  |  |  |  |  |  |  |  |
| 7: Explotación de resultados e impacto económico-social | | | |  |  |  |  |  |  |  |  |  |  |  |  |  |  |  |  |  |  |  |  |  |  |  |  |  |  |  |  |  |  |  |  |  |  |  |  |
| 8: Coordinación y dirección científico-técnica | | |  |  |  |  |  |  |  |  |  |  |  |  |  |  |  |  |  |  |  |  |  |  |  |  |  |  |  |  |  |  |  |  |  |  |  |  |  |

HITO 4

HITO 6

HITO 8

# Evaluación del proyecto

## Metodología de evaluación

La evaluación se puede plantear en dos fases: una primera experiencia piloto con diez pacientes por rama, que se evaluará tras seis meses de seguimiento, y una evaluación posterior alcanzando un total de 100 pacientes (50 en cada rama) durante 2 años.

Hay múltiples herramientas disponibles para realizar la evaluación. Algunas son:

- Método de “input logging”: utiliza un sistema de registro automático de acciones de usuario integrado en el sistema de información.
- Método de observación directa: consiste en seleccionar, registrar y codificar un conjunto de conductas del usuario, mediante observaciones directas del evaluador.
- Método de cuestionarios: solicita a los usuarios que rellenen un cuestionario relativo a las acciones efectuadas, sus expectativas, actitudes y opiniones de todo tipo sobre el sistema.
- Método de entrevista: realizar al usuario un conjunto de preguntas en los mismos aspectos del método anterior.

La evaluación de un programa de telemedicina requiere la definición y aplicación de todo este conjunto de métodos de evaluación realizando este proceso desde diferentes puntos de vista. Es decir, los objetivos de la evaluación deben incluir aspectos técnicos, clínicos y económicos, sin olvidar los referentes a la organización.

### Evaluación técnica

Esta evaluación se llevará a cabo de dos formas distintas, según el momento de aplicación. Será *formativa* durante las etapas iniciales de desarrollo del sistema con el fin de mejorar y corregir sus errores (tanto técnicos como de usabilidad). Y se realizará una evaluación *sumativa* al finalizar el desarrollo del sistema. La evaluación técnica debe contemplar tanto la validación de la funcionalidad del sistema, como la caracterización de los procedimientos de cuidado telemédicos para su integración en rutina clínica y por último la evaluación de la usabilidad del sistema.

- Validación técnica

La validación técnica del sistema sirve para comprobar que el sistema cumple con las funcionalidades especificadas en su momento.

- Caracterización de protocolos de telemedicina

La evaluación de los protocolos de telemedicina tiene por objetivo analizar los nuevos procedimientos generados por el uso de la telemedicina en la práctica clínica e identificar las características del sistema más adecuadas para una posterior implantación a gran escala. La caracterización de los protocolos de telemedicina se obtiene mediante el registro de los perfiles de uso del sistema de telemedicina identificando parámetros clave como la frecuencia de uso, la optimización de visitas al médico, uso de los diferentes servicios ofrecidos,... Este registro se podría realizar mediante técnicas de web mining, facilitando enormemente su análisis posterior.

- Evaluación de usabilidad

Se evaluarán la utilidad, eficiencia, facilidad de uso y de aprendizaje, etc. Para ello se emplearán los métodos anteriormente comentados.

### Evaluación clínica

El objetivo principal de esta evaluación es estudiar si el sistema mejora la calidad asistencial al enfermo infectado por el VIH, respecto al control habitual que realiza, y su viabilidad en rutina clínica. También es importante valorar el impacto del sistema en la calidad de vida del paciente.

Algunos de los criterios de valoración para la evaluación clínica serán:

- Control de la infección por VIH, medido por respuesta virológica e inmunológica al tratamiento antirretroviral, fracasos de la medicación, desarrollo de resistencias y, en su caso, infecciones oportunistas y/o muerte.
- Medición de adherencia a la medicación antes y después del estudio.
- Efectos secundarios de la medicación.
- Impacto en la calidad de vida medida a través de cuestionarios.
- Encuestas de satisfacción antes, durante y después del estudio.
- Tiempo de espera de las visitas y número de visitas necesario.

### Evaluación económica y de organización

Que tiene como objetivo principal analizar la relación coste-beneficio en la introducción del sistema en rutina clínica, así como el impacto del sistema en la organización clínica, comparando cómo se proveía el servicio con y sin la telemedicina.

Algunos de los parámetros económicos a tener en cuenta serán:

- Coste en medicamentos antes y después del estudio.
- Parámetros farmacoeconómicos.
- Coste total del control del paciente en un año.

# Aparición de acontecimientos adversos

## Información mínima a especificar

Descripción/ definición: Acontecimiento adverso es cualquier experiencia no deseada que ocurra a un sujeto durante su participación en un ensayo clínico, se considere o no relacionada con el producto o productos en experimentación. Reacción adversa es cualquier efecto perjudicial o indeseado que se presente tras la administración de un medicamento a las dosis normalmente utilizadas en el hombre para la profilaxis, diagnóstico o tratamiento de una enfermedad.

Gravedad: Los acontecimientos graves son los que determinan el fallecimiento del sujeto, riesgo de fallecimiento, incapacidad permanente o significativa, hospitalización o prolongación de la hospitalización, o anomalías congénitas o procesos neoplásicos. (Se entiende por "riesgo de fallecimiento" la situación en que, en opinión del médico, de no haber mediado una intervención terapéutica oportuna, se hubiera producido el fallecimiento del paciente.)

Los acontecimientos adversos inesperados son aquellos que no se encuentran descritos en la información básica del producto, en cuanto a naturaleza, gravedad o frecuencia.

Método de detección y registro: los acontecimientos adversos se recogerán a partir de la cuidadosa observación clínica del paciente, análisis de laboratorio, comunicación espontánea del paciente, y también mediante un interrogatorio abierto.

De cada acontecimiento se recogerá su intensidad, duración, relación temporal con la administración del fármaco, necesidad de tratamiento y posibles causas alternativas.

## Criterios de imputabilidad

A fin de analizar la posible relación causa-efecto, se detallará la fecha de inicio y remisión, las medidas terapéuticas adoptadas -ninguna, interrupción del tratamiento, tratamiento específico, la evolución -remisión completa, secuelas, persistencia-, persistencia o no tras la suspensión de la administración, o reaparición con la readministración del producto.

Para evaluar la imputabilidad se usará el algoritmo de Karch y Lasagna modificado.

## Procedimientos para la notificación inmediata de los acontecimientos adversos graves o inesperados

El investigador principal comunicará los acontecimientos adversos al Centro Coordinador, quien a su vez, informará a las autoridades sanitarias. Esta comunicación no exime al investigador de la responsabilidad de informar al Comité Ético de Investigación Clínica (CEIC) correspondiente.

Se comunicará todo acontecimiento adverso que se detecte durante el desarrollo del ensayo clínico.

Aquellos acontecimientos adversos que sean mortales, o conlleven riesgo vital, se comunicarán en el plazo de 24 horas al coordinador del estudio.

Los acontecimientos adversos que, aunque no entrañen riesgo vital, sean graves o inesperados, se comunicarán en el formulario de notificación en el plazo de 15 días.

La información sobre acontecimientos adversos que no sean graves o inesperados, se recogerá en forma tabulada al final del ensayo clínico o coincidiendo con los análisis intermedios cuando éstos estuvieran previstos.

La información sobre acontecimientos adversos, recogida según las normas previas, se remitirá a la Agencia Española del Medicamento (subdirección de Medicamentos de Uso Humano).

# Incumplimientos del protocolo

## Abandono del tratamiento, negativa a seguir

Si un paciente se niega a seguir, se le podrá intentar cambiar al control habitual.

## Aparición de un embarazo

En este supuesto, se cambiará a control habitual de personas embarazadas y saldrá del estudio.

# Medidas de seguridad

También es importante destacar los aspectos ético-legales, debido a la confidencialidad de los datos clínicos, habrá un comité de seguridad independiente que controlará en todo momento el estudio.

## Ética y confidencialidad de los datos.

### Aprobación del estudio por el Comité de Etica y de Investigación Clínica

El estudio es aprobado por el Comité de Etica e Investigación Clínica del hospital.

### Confidencialidad de los datos.

Los cuadernos de observación y los registros de los análisis biológicos realizados en el marco de este estudio quedarán identificados sólo con el número de identificación asignado a cada paciente en el estudio.

Todos los documentos se conservarán en un local cerrado con llave.

Los ficheros informáticos se constituirán de conformidad con la ley sobre Informática y Libertad.

# Infraestructura

Como infraestructura en el hospital se utilizaría una consulta determinada para estos pacientes. Se necesitaría un servidor y 4 ordenadores conectados en red, con conexión a Internet y cámaras para videoconferencia, además de 5 agendas PDA para la comunicación y coordinación de los profesionales.

En los domicilios de los pacientes se deberá disponer de conexión a Internet, por lo que si estos no poseen un ordenador de características suficientes, se les dotará de un terminal de conexión a Internet alternativo con su correspondiente cámara de videoconferencia y el acceso ADSL. Este acceso a Internet podría ser desde un ordenador normal, desde un home hub (ordenador con apariencia de video conectado al televisor del domicilio) o desde un terminal de conexión a Internet.

En el laboratorio donde se realizará el desarrollo del sistema se necesita duplicar el sistema (exacto) que se instalará en el hospital y en los domicilios para realizar las correspondientes pruebas y poder subsanar errores. Por ello, se le dotará de los equipos correspondientes.

# Presupuesto

El estudio será financiado en su totalidad por becas, apoyos privados, subvenciones públicas... Para mayor comodidad se ha dividido el presupuesto en los dos grupos de investigación involucrados en el proyecto durante sus tres años de duración:

**ANEXO I**

**INDICE DE KARNOFSKY**

100% Normal, sin indicios de enfermedad.

90% Vida normal, signos o síntomas menores de la enfermedad.

80% Actividad normal con esfuerzo, algunos signos o síntomas menores de la enfermedad.

70% Puede arreglárselas solo; incapaz de desarrollar actividades normales o de trabajar.

60% Necesita ayuda ocasionalmente, pero puede arreglárselas solo en la mayoría de las situaciones.

50% Necesita ayuda regularmente y cuidados médicos frecuentes.

40% Disminuido, necesita ayuda y cuidados especiales.

30% Muy disminuido, está indicada la hospitalización, aunque la muerte no es inminente.

20% Hospitalización necesaria, muy enfermo, necesita un tratamiento de apoyo activo.

10% Moribundo, proceso fatal que progresa rápidamente.

**ANEXO II**

**Estudio piloto de la viabilidad de un hospital real/virtual en el Hospital Clínic de Barcelona para enfermos infectados por el VIH estables, en estadío crónico de su enfermedad.**

**NOTA INFORMATIVA**

Por favor, lea atentamente este documento informativo

*INTRODUCCION*

Actualmente, la telemedicina y la informática médica, pueden ayudar a un mejor control de los pacientes. El Servicio de Telemedicina es una herramienta que debería servir no para sustituir a la relación clásica médico-paciente (como se plantea en algunos sitios de hospitales únicamente virtuales), sino para mejorar esta relación, haciendo que el paciente participe en su propio control (como ya se hace con éxito en otras enfermedades crónicas como la diabetes). Esta mayor implicación del paciente en su enfermedad requiere una disponibilidad de información, y autogestión que solo la informática nos puede ofrecer.

Muy probablemente esta implicación conlleve una mayor satisfacción del paciente, pero además debe conllevar un mejor control del mismo, asegurando las tomas de medicación, minimizando los efectos secundarios

*OBJETIVOS DEL ESTUDIO*

Estudiar si un servicio de telemedicina mejora la calidad asistencial (medida por la satisfacción del paciente, la adherencia a la medicación, el control médico del paciente, la disminución de efectos secundarios de la medicación, tiempo de espera de las visitas, el acceso a información, el acceso a atención especializada) respecto al control habitual que realizan los enfermos.

*DESCRIPCION DEL ESTUDIO*

A pacientes, que como usted, cumplen los criterios de estar en tratamiento antirretroviral de forma estable se les propondrá comenzar un estudio de control a través de telemedicina alternado con controles reales en su consulta.

Podrá realizar desde casa toda una serie de funciones que se detallan:

- Gestión de las consultas virtuales: bien de enfermería, para consultas sobre educación, preventivas; médicas, para consultas concretas sobre efectos secundarios, dudas, resultados de pruebas; bien para el equipo de atención psicológica o social.
- Gestión de la medicación: solicitud automática de medicamentos al servicio de enfermería y envío a domicilio, así como la notificación de cambios en la terapia. También se dispondrá de herramientas de monitorización del tratamiento (cumplimiento, efectos secundarios,...).
- Gestión de la información: disponibilidad de acceso ilimitado a una información elaborada para pacientes sobre la enfermedad, medicamentos, efectos secundarios, posibilidades de futuro, ensayos clínicos, innovaciones, acceso a recursos de la zona, noticias...
- Gestión de la comunidad virtual: participación del paciente en una comunidad virtual con chat para compartir experiencias con otros enfermos.

El seguimiento será de dos años de duración.

Será usted controlado con 2 visitas reales y 2 virtuales. Los análisis y la medicación seguirán realizándose igual que en la actualidad.

*CONFIDENCIALIDAD*

Cualquier información referida a usted será estrictamente confidencial y quedará protegida por el secreto médico. Todos los datos que se recojan se protegerán con el anonimato antes de procesarlos informáticamente.

Su consentimiento para participar en este estudio supone que acepta usted que todos los médicos o científicos que intervengan en él tengan acceso a la información que figura en su expediente, siempre dentro de la más estricta confidencialidad. Acepta usted asimismo que los datos científicos sean procesados informáticamente, información ésta que quedará reservada para uso exclusivo del médico metodólogo, del médico investigador principal del estudio y de sus respectivos equipos.

En caso de que sufriera algún perjuicio por la participación en el estudio, se pondrán en marcha a su disposición todos los medios disponibles para subsanarlo. Existe además un seguro de responsabilidad civil que cubriría tal eventualidad. La firma de la hoja de consentimiento no supone la renuncia a ningún derecho.

Por último, su médico le comunicará personalmente los resultados de este estudio en cuanto estén disponibles.

**ANEXO III**

**FORMULARIO DE CONSENTIMIENTO INFORMADO**

D. (apellidos, nombre)..............................................................................................................

El Dr. ............................... me ha propuesto participar en el estudio descrito en la nota informativa.

- He leído y comprendido la nota informativa que me ha sido facilitada a propósito de este estudio.

- He discutido de ello con el Dr. ................................., quien me ha explicado con detalle la naturaleza, los objetivos y los riesgos potenciales de mi participación en el estudio.

- Se me ha garantizado que las decisiones que se adopten relativas a mi salud se tomarán en todo momento de acuerdo con el estado de los conocimientos acerca de la infección por el VIH y el tratamiento antirretroviral.

- He comprendido que soy libre de aceptar o de negarme a participar en el estudio y, si aceptara, de interrumpir mi participación en cualquier momento sin ningún temor, aunque me comprometo en tal caso a ponerlo previamente en conocimiento del Dr...............................

- Doy mi autorización para que los datos confidenciales referidos a mi persona puedan ser consultados por las personas que intervienen en el estudio y, eventualmente, por representantes de las autoridades sanitarias.

- Estoy informado que este estudio ha recibido un dictamen favorable emitido por el Comité de Etica y de Investigación Clínica.

- En caso de solicitarlo, los resultados de mis exámenes y los del estudio en general me serán comunicados por mi médico.

Acepto libremente participar en este estudio de investigación en las condiciones mencionadas en la nota informativa y en el presente documento.

Dado en ....................................., ........ de ........................... de ..............

Firma

El abajo firmante, Doctor ........................................., responsable del centro, certifica que ha facilitado o hecho llegar al Sr. D. ............................................................. toda la información útil referida a los objetivos y las modalidades de este estudio, y se compromete a que el centro respete los términos del presente formulario de consentimiento con objeto de que esta investigación se desarrolle en las mejores condiciones, compaginando el respeto de los derechos y las libertades individuales con las exigencias de un trabajo científico.

Dado en ................................, ............ de............................... de.............

Firma del médico

**ANEXO IV: Hoja de notificación de acontecimientos adversos.**

**INSTRUCCIONES GENERALES**

**1.** Este formulario se utilizará solamente para comunicar los acontecimientos adversos mortales, graves o inesperados que ocurran durante el desarrollo del ensayo clínico.

**2.** Los acontecimientos adversos mortales o que entrañen riesgo vital (aquellos que de no haber mediado una intervención terapéutica inmediata hubieran supuesto la muerte del paciente) se comunicarán en el plazo máximo de 72 horas; si no se dispusiera de toda la información, esta podrá complementarse en el plazo de 15 días. Los demás acontecimientos adversos graves o los inesperados se comunicarán en el plazo de 15 días.

**3.** Cuando el espacio disponible sea insuficiente, se añadirá una hoja de información adicional, correctamente identificada con el nombre del promotor y el número asignado a la notificación. En esta información adicional podrá hacerse constar la evaluación de la causalidad realizada por el Técnico que informa.

**INSTRUCCIONES PARA RELLENAR CORRECTAMENTE ESTA HOJA**

**1.** El número de protocolo es el código alfanumérico específico asignado por el promotor.

El número de notificación del promotor es el que éste utiliza para su archivo. Cuando se trate de información de seguimiento se utilizará el mismo número o bien, si se modifica, se indicará el número de la notificación inicial. Se dejará sin rellenar el espacio "Nº de notificación" que aparece sombreado.

**2.** Las iniciales del paciente (o del profesional sanitario que detectó la R.A.) seguirán las siguientes normas:

Las dos primeras posiciones por la izquierda para las iniciales del primer y segundo nombre y las dos últimas para las del primer y segundo apellido.

Poner un 0 en la primera posición si sólo se tiene un nombre, que se situará en la segunda posición. En caso de apellidos compuestos utilizar sólo las iniciales del primer componente del apellido compuesto. Ejemplo:

JLPG Jose Luis Pérez González

0JPG Jose Pérez González

JLPR Jose Luis Pérez-González y Rodríguez

0JPR Jose Pérez-González y Rodríguez-Gómez

Al codificar apellidos, se prescindirá de preposiciones y artículos.

**3.** La edad se pondrá en años, meses, semanas o días, según convenga.

4**.** Se describirá el acontecimiento adverso en forma completa, indicando la fecha de finalización del mismo e incluyendo los resultados de las exploraciones complementarias o pruebas de laboratorio que se consideren de interés. A esta notificación podrán acompañarse cuantos informes se estimen convenientes para la adecuada interpretación del cuadro clínico.

5**.** Los productos en fase de investigación se identificarán a ser posible por su nombre genérico (DCI) o, en su defecto, por el código de investigación del producto. Se considera producto de investigación tanto el producto específicamente investigado como el control.

6**.** En caso de que la administración no sea diaria se intentará describirla con alguna de las siguientes posibilidades: cíclica, semanal, mensual, anual o número de veces que se ha utilizado (poniendo en este caso la dosis de cada toma, no la total).

7**.** Se hará constar el proceso patológico del paciente al que va destinado el producto en investigación o bien "voluntario sano" en caso de tratarse de tal.

8**.** Se hará constar la duración del tratamiento hasta el inicio del acontecimiento adverso.

| **NOTIFICACIÓN DE ACONTECIMIENTOS ADVERSOS** | PROTOCOLO Nº: | Nº DE NOTIFICACIÓN:  (laboratorio) |
| --- | --- | --- |
| PRODUCTO EN FASE DE INVESTIGACIÓN CLÍNICA | PACIENTE Nº: | Nº DE NOTIFICACIÓN: |

**I. INFORMACIÓN SOBRE EL ACONTECIMIENTO ADVERSO**

| 1. INICIALES DEL PACIENTE | 1a. PAIS | 2. FECHA DE NACIMIENTO | | | 2a. EDAD | 3. SEXO | 3.a PESO | | 4-6 INICIO | | |
| --- | --- | --- | --- | --- | --- | --- | --- | --- | --- | --- | --- |
|  |  | DIA | MES | AÑO |  |  |  | | DIA | MES | AÑO |
| 7. DESCRIPCIÓN DEL ACONTECIMIENTO ADVERSO (Incluyendo resultados relevantes de exploraciones o de laboratorio) | | | | | | | | 8-13 CONSECUENCIAS   FALLECIMIENTO   LA VIDA DEL PACIENTE HA ESTADO EN PELIGRO   HOSPITALIZACIÓN O PROLONGACIÓN DE LA HOSPITALIZACIÓN   INCAPACIDAD PERMANENTE O SIGNIFICATICVA   PERSISTENCICA DEL ACONTECIMIENTO ADVERSO   RECUPERACIÓN | | | |

**II INFORMACIÓN DEL PRODUCTO EN INVESTIGACIÓN**

| 14 NOMBRE | |
| --- | --- |
| 15 DOSIS DIARIA | 16. VIA DE ADMINISTRACIÓN |
| 17. ENFERMEDAD EN ESTUDIO | |
| 18. FECHAS DEL TRATAMIENTO (Desde/Hasta) | 19. DURACIÓN DEL TRATAMIENTO |
| 20. ¿REMITIÓ EL ACONTECIMIENTO AL SUSPENDER LA MEDICACIÓN?   SI  NO  NO PROCEDE | |
| 21. ¿REAPARECIÓ EL ACONTECIMIENTO AL ADMINISTRAR DE NUEVO LA MEDICACIÓN?   SI  NO  NO PROCEDE | |

**III. HISTORIA CLÍNICA Y MEDICAMENTOS CONCOMITANTES**

| **¡Error! Marcador no definido.22. MEDICAMENTOS CONCOMITANTES Y FECHA DE ADMINISTRACIÓN** |
| --- |
| **23. DATOS IMPORTANTES DE LA HISTORIA CLÍNICA (ej. diagnóstico, alergias, embarazo, enfermedades, concomitantes, etc.)** |

***IV. INFORMACIÓN SOBRE PROMOTOR E INVESTIGADOR***

| ¡Error! Marcador no definido.**24a. NOMBRE Y DIRECCIÓN DEL PROMOTOR** | | **24b. NOMBRE Y DIRECCIÓN DEL INVESTIGADOR** |
| --- | --- | --- |
| **24d. CÓDIGO DEL LABORATORIO**  **(Nº DGFPS)** | **25a. TIPO DE INFORME**   **INICIAL**   **SEGUIMIENTO** | **24c. NOMBRE DEL TÉCNICO QUE INFORMA**  **NOMBRE:**  **TFNO.:**  **FIRMA:** |
| **24e. FECHA DEL INFORME** | **24f. FECHA DE ENTRADA DGFPS** | **25b.**  **SE ADJUNTA INFORME COMPLEMENTARIO** |
